# The efficacy of transcranial magnetic stimulation (TMS) for negative symptoms in schizophrenia: a systematic review and meta-analysis

**DOI:** 10.1038/s41537-022-00248-6

**Published:** 2022-04-09

**Authors:** Rasmus Lorentzen, Tuan D. Nguyen, Alexander McGirr, Fredrik Hieronymus, Søren D. Østergaard

**Affiliations:** 1 Department of Affective Disorders, Aarhus University Hospital – Psychiatry, Aarhus, Denmark; 2 Department of Clinical Medicine, Aarhus University, Aarhus, Denmark; 3 Hotchkiss Brain Institute, University of Calgary, Calgary, Canada; 4 Department of Psychiatry, Cumming School of Medicine, University of Calgary, Calgary, Canada; 5 Mathison Centre for Mental Health Research and Education, University of Calgary, Calgary, Canada; 6 Department of Pharmacology, Sahlgrenska Academy, University of Gothenburg, Gothenburg, Sweden

**Keywords:** Schizophrenia, Neural circuits

## Abstract

Several trials have shown preliminary evidence for the efficacy of transcranial magnetic stimulation (TMS) as a treatment for negative symptoms in schizophrenia. Here, we synthesize this literature in a systematic review and quantitative meta-analysis of double-blind randomized controlled trials of TMS in patients with schizophrenia. Specifically, MEDLINE, EMBASE, Web of Science, and PsycINFO were searched for sham-controlled, randomized trials of TMS among patients with schizophrenia. The effect of TMS vs. sham on negative symptoms in each study was quantified by the standardized mean difference (SMD, Cohen’s *d*) with 95% confidence intervals (95%CI) and pooled across studies using an inverse variance random effects model. We identified 57 studies with a total of 2633 participants that were included in the meta-analysis. The pooled analysis showed statistically significant superiority of TMS (SMD = 0.41, 95%CI: 0.26; 0.56, *p*-value < 0.001), corresponding to a number needed to treat of 5. Furthermore, stratified analyses suggested that TMS targeting the left dorsolateral prefrontal cortex and using a stimulation frequency >1 Hz was most efficacious. There was, however, substantial heterogeneity and high risk of bias among the included studies. In conclusion, TMS appears to be an efficacious treatment option for patients with schizophrenia suffering from negative symptoms, but the optimal TMS parameters are yet to be established.

## Introduction

Pharmacological treatment is the cornerstone of care in schizophrenia and other psychotic disorders^1^. Though positive symptoms (e.g., delusions and hallucinations) respond relatively well to pharmacological treatment, negative symptoms often do not respond to the same degree^2–4^. The negative symptoms of schizophrenia are those representing absence/lessening of normal functions and include affective flattening, alogia, apathy and social withdrawal^5^. Patients with predominantly negative symptoms are more resistant to treatment than patients with primarily positive symptoms, and negative symptoms are strongly associated with low daily functioning and poor long-term prognosis^6–8^. Therefore, identification and development of efficacious treatments of negative symptoms is a priority^3,9^.

Transcranial magnetic stimulation (TMS) is a non-invasive neuromodulation technique in which a localized electrical field is elicited in underlying brain parenchyma through electromagnetic induction, generally limited to superficial cortical regions^10,11^. Whether TMS increases or decreases the activity of the targeted neurons depends on the frequency of the magnetic pulses with 1 Hz and below (low frequency) being inhibitory and >1 Hz being excitatory (high frequency)^12^. Repetitive TMS (rTMS) is the most widely used modality with a single session lasting 20–40 min, typically delivering between 1200-3000 magnetic pulses^13^. Other types include deep TMS in which the magnetic field reaches deeper subcortical regions of the brain as well as theta burst stimulation (TBS) in which the frequency of stimulation is 50 Hz administered five times per second to mimic endogenous theta waves either continuously or intermittently^14^.

TMS is approved for the treatment of major depression (https://www.accessdata.fda.gov/cdrh_docs/pdf6/K061053.pdf) and since the negative symptoms of schizophrenia and the symptoms of major depression both represent deficits of normal functions, TMS has also been explored as a potential treatment of negative symptoms among patients with schizophrenia^15–18^. Since the most recent reviews of the literature on TMS for treatment of negative symptoms^15–18^, several trials have been conducted—some using novel stimulation parameters as well as neuronavigation to improve targeting^19–27^. Due to these developments in the field, an updated synthesis would be of relevance. Therefore, we conducted a systematic review and quantitative meta-analysis of randomized controlled trials reporting on the efficacy of rTMS in the treatment of negative symptoms among patients with schizophrenia.

## Methods

### Protocol and registration

The study protocol was registered at the International Prospective Register of Systematic Reviews (PROSPERO, ID: CRD42021238828) (https://www.crd.york.ac.uk/PROSPERO) and carried out in accordance with the Preferred Reporting Items for Systematic Reviews and Meta-Analyses (PRISMA) guidelines^28^.

### Information sources and screening

MEDLINE (PubMed), PsycINFO, Web of Science and EMBASE were searched for relevant studies. Earlier reviews on the subject, clinicaltrials.gov, as well as citations of included studies were reviewed in order to find further eligible studies. The search was carried out on May 1^st^ 2021 using the following search string in MEDLINE: (“schizophreni*” OR “schizoaffective disorder” OR “schizophreniform disorder” OR “schizophrenia”[MeSH Terms] OR “negative symptom*” OR “CHR” OR “Clinical High Risk” OR “Ultra High Risk” OR “UHR” OR “Psychotic Disorders”[MeSH Terms] OR “Psychotic Disorder*“) AND (“transcranial magnetic stimulation” OR “TMS” OR “rTMS” OR “theta burst” OR “iTBS” OR “cTBS” OR Transcranial Magnetic Stimulation*[MeSH Terms]). The analogue search strings used for the other databases are available in the Supplementary Material. Titles and abstracts of studies identified via the search strategy described above were screened independently by two authors (RL and TDN) assisted by Covidence^29^. Full text versions of the studies deemed relevant after initial screening were subsequently assessed for eligibility.

### Eligibility criteria

In line with earlier reviews in the field, the following inclusion criteria were employed^15,17,18^. Notably, no language restrictions were employed:

- Randomized, sham-controlled trials of transcranial magnetic stimulation (e.g., rTMS or theta burst stimulation)
- Participants with a primary diagnosis of schizophrenia, schizoaffective disorder or another psychotic disorder (e.g., acute/transient/brief psychotic disorder or persistent delusional disorder), according to the DSM-IV, DSM-5, or ICD-10.
- Adult participants (≥18 years)
- Outcome measured using an established psychometric scale for negative symptoms in schizophrenia (e.g., the negative subscale of the Positive and Negative Syndromes Scale (PANSS-N)^30^ or the Scale for Assessment of Negative Symptoms (SANS)^31^).

The following exclusion criterion was employed:

- Co-initiation of other treatments, e.g., pharmacological treatment, as the results of such studies could be affected by an interaction effect between TMS and the co-initiated treatment.

### Data extraction

The following items were extracted from each included study: Author name, publication year, country, study type (cross-over or parallel), analysis-type (per protocol or intention-to-treat (ITT)), number of participants, drop-out rates, mean age of participants, sex distribution of participants, diagnostic distribution, whether samples were selected for predominantly negative symptoms, frequency and intensity of TMS including the total number of stimuli and number of treatments, TMS target, nature of the sham intervention, outcome measure (rating scale), post treatment scores, follow-up scores and post treatment depression scores (if available). If these data were not reported, the authors were contacted by e-mail with a request to provide the data. If authors did not reply, data from graphs (if available) were extracted using the GetData Graph Digitizer (http://getdata-graph-digitizer.com/). Previous meta-analyses were screened for post-treatment outcome data required to compute effect sizes. Studies where data was not available upon request, via graphs or through previous meta-analyses, were excluded from the analyses.

### Evaluation of risk of bias

The included studies were evaluated according to five domains of bias (articles in non-English languages were not evaluated) using the Cochrane Risk of Bias Tool 2.0:(https://methods.cochrane.org/risk-bias-2) (A) Randomization process (allocation sequence generation and concealment), (B) Deviations from intended interventions (bias arising from non-protocol interventions), (C) Missing outcome data (dropouts), (D) Measurement of the outcome (using a validated tool), and (E) Selection of the reported result (alignment with protocol and method section). In accordance with the instructions for the Cochrane Risk of Bias Tool 2.0, the highest risk score assigned in one of these domains defined the overall risk of bias score for each study (https://methods.cochrane.org/risk-bias-2). Furthermore, potential publication bias was explored via a funnel plot and Egger’s regression test.

### Statistical analysis

The effect of TMS vs. sham on negative symptoms in each included study was quantified by the standardized mean difference (SMD, Cohen’s *d*) with 95% confidence intervals (95%CI) based on endpoint scores or change scores (with endpoint scores being preferred). If multiple outcome measures were used, PANSS-N was preferred to improve methodological homogeneity because it was used in 89% (51/57) of the included studies. If a study did not provide standard deviations (SD) or data that could be used to calculate SD (e.g., standard error), the mean standard deviation across all studies of the same outcome measure was used. For cross-over studies, data was extracted after the first treatment phase (before cross-over) to exclude possible carry-over effects of treatment and thus regarded as a parallel design study.

SMDs were pooled using the inverse variance random effects model in Review Manager 5.3^32^. This model takes into account both in-study and between-study variability. For the primary analysis, number needed to treat (NNT) was estimated using the method proposed by Kraemer and Kupfer^33^. Heterogeneity was assessed using the *I*²-test with *I*²-values ≥50% suggesting considerable heterogeneity. For multi-arm studies, data from different active TMS treatment arms were pooled in the calculation of overall efficacy as to not duplicate data from the sham group, using the formulas provided in table 6.5a in the Cochrane Handbook (https://training.cochrane.org/handbook/current/chapter-06#section-6-5-2-10).

Following the main analysis, the following secondary/subgroup analyses (yielding effect sizes) were carried out: (i) focusing on long term effect using data from at least four weeks after the last treatment (the last follow up in each study was used), (ii) focusing on patients with predominantly negative symptoms, (iii) focusing on the effect size of TMS for depressive symptoms (all depression measures allowed with a preference for the Calgary Depression Scale in Schizophrenia^34^), (iv) after stratifying by target site (v) after stratifying by type of TMS, (vi) after stratifying by stimulation frequency, (vii) after stratifying by stimulation intensity, and (viii) after stratifying by age. Finally, the following sensitivity analyses (yielding effect sizes) were conducted: (I) after excluding studies with data extracted from graphs, (II) after excluding studies with high risk of bias, (III) after excluding studies reporting change-from-baseline scores, (IV) after excluding studies with data extracted from other reviews, and (V) after stepwise exclusion of the 10 most outlying studies compared to the overall efficacy estimate.

## Results

### Study selection

**Fig. 1:**
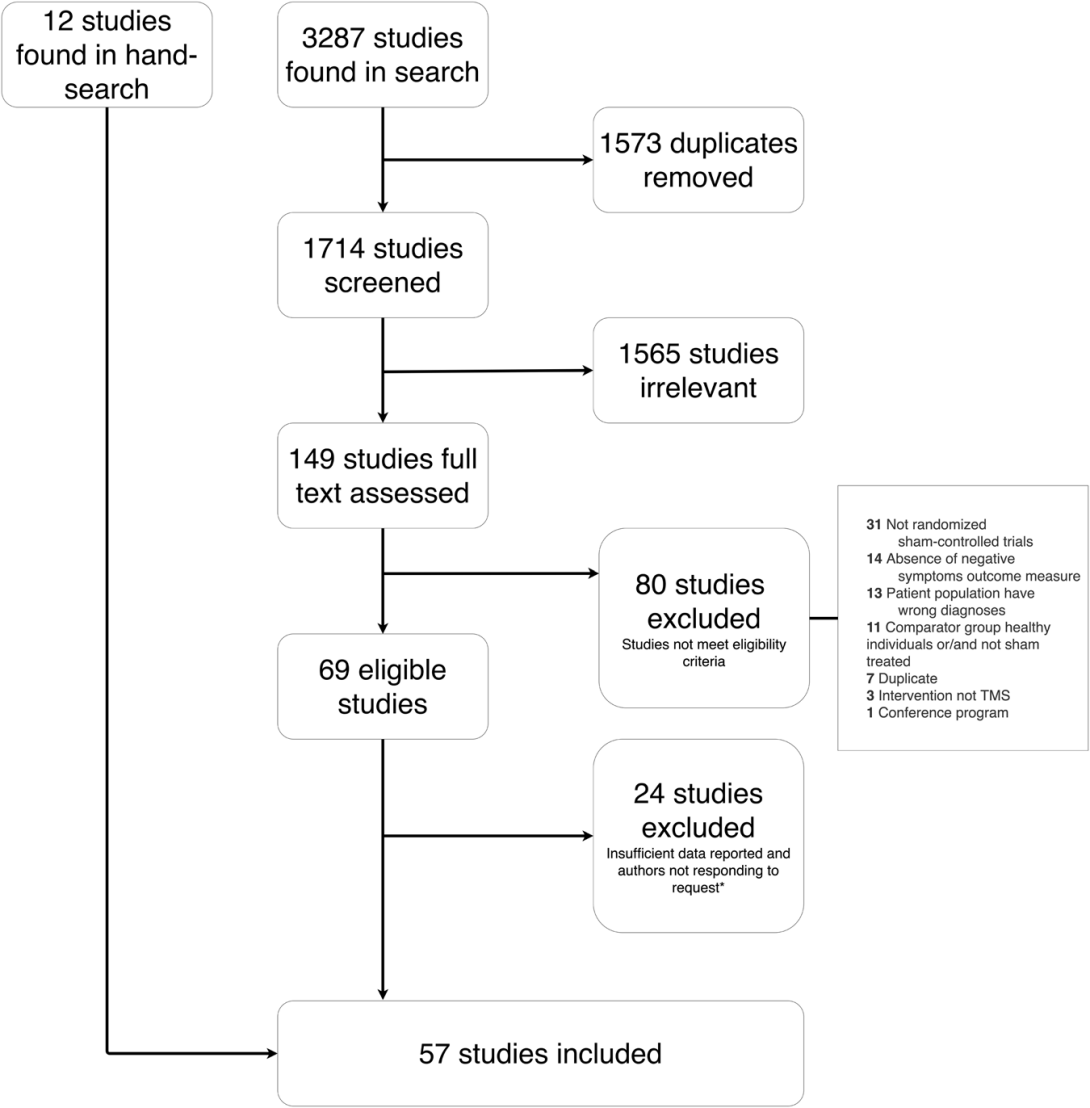
PRISMA flowchart illustrating the literature screening. *Authors were contacted by e-mail. If data was not provided and data could not be taken from graphs, the study was excluded.

The search yielded 3287 articles of which 1573 were duplicates, resulting in 1714 studies that underwent title- and abstract screening (Fig. 1). Following this screening, 1565 were excluded. This left 149 articles to be assessed in full text, of which 80 studies did not meet the eligibility criteria. The most common reason for exclusion in this final step was “not randomized sham-controlled trials” (31 studies), which included non-blinded studies, studies with no sham-treated group and studies, which were not clinical trials. The second most common reason for exclusion was “did not include eligible outcome measure” (14 studies), which included studies that did not measure negative symptoms, but had other primary endpoints such as biomarkers/neuroimaging or cognitive function. Of the 69 eligible studies, 35 reported insufficient data and thus the authors were contacted by e-mail requesting additional data. The means and standard deviations of PANSS-N was the most common missing piece of information (i.e., from studies where only the total PANSS scores were reported). From these 35 studies, three author groups provided data^21,26,35^, and data were extracted from graphs in an additional eight studies^27,36–42^. Hence, 24 articles were excluded due to non-available data^43–66^. In total, the search yielded 45 includable studies, with 51 comparisons as a result of studies including multiple interventions^19–27,35–42,67–94^. No additional studies were found in citations or in the database of clinicaltrials.gov. Summary data was available from 15 studies reviewed by Wang and colleagues^18^ from non-English reports^90–92,95–106^, of which 3 were also identified by the database search, leaving a total of 57 studies and 63 comparisons^19–27,35–42,66–106^.

### Study characteristics

Table 1 shows the characteristics of the 57 included studies.

**Table 1:** Characteristics of the included studies.

Author	Year	Country	N_a_	N_c_	Mean age	PNS	Analysis	Study type	% males	Drop-out rate	Outcome score	TMS type	Sham type	Hz	% MT	Treatment sessions	Total number of stimuli	Brain target
Bai	2015	China	36	35	34.9	NA	NA	NA	NA	NA	PANSS-N	rTMS	NA	10	100%	NA	25,000	L-DLPFC
Bais	2014	Netherlands	31	16	36.2	N	ITT	Par	59%	9%	PANSS-N	rTMS	Sham coil	1	90%	12	14,400	B-TPC, L-TPC
Barr	2012	Canada	13	12	45.4	N	PP	Par	68%	24%	PANSS-N, SANS	rTMS	90°	20	90%	20	30,000	B-DLPFC
Bation	2021	France	12	10	35.5	Y	ITT	Par	95%	0%	SANS, PANSS-N	iTBS	Sham coil	50	80%	20	19,800	L-DLPFC
Chauhan	2020	India	19	17	40.2	N	ITT	Par	42%	17%	PANSS-N	iTBS	Sham coil	50	80%	10	12,000	MC
Chen	2011	China	24	22	38.5	NA	NA	NA	NA	NA	PANSS-N	iTBS	NA	NA	80%	20	48,000	L-DLPFC
Chibbaro	2005	Italy	8	8	40.4	N	NA	Par	69%	NA	SANS	rTMS	45°	1	90%	4	3600	L-TPC
Cordes	2010	Germany	12	13	34.4	Y	PP	Par	100%	4%	PANSS-N	rTMS	Sham coil	10	110%	10	10,000	L-DLPFC
de Jesus	2011	Brazil	8	9	39.5	N	ITT	Par	71%	0%	BPRS-N/D	rTMS	45°	1	90%	20	23,040	L-TPC
Dlabac-de Lange	2014	Netherlands	16	16	35.5	Y	ITT	Par	81%	0%	PANSS-N, SANS	rTMS	90°	10	90%	30	60,000	B-DLPFC
Dollfus	2018	France	26	33	38.3	N	PP	Par	54%	20%	PANSS-N	rTMS	Sham coil	20	80%	4	10,400	L-STS
Duan	2013	China	21	20	26.9	NA	NA	NA	NA	NA	PANSS-N	rTMS	NA	10	100%	20	NA	L-DLPFC
Fitzgerald	2008	Australia	12	8	34.7	Y	ITT	Par	80%	25%	PANSS-N, SANS	rTMS	90°	10	110%	15	30,000	B-PFC
Gan (a)	2014	China	20	21	26.9	NA	NA	NA	NA	NA	PANSS-N	TBS	NA	NA	100%	20	NA	L-DLPFC
Gan (b)	2014	China	38	37	27.1	NA	NA	NA	NA	NA	PANSS-N	rTMS	NA	10	100%	20	NA	L-DLPFC
Gan	2015	China	32	35	28.5	NA	NA	NA	NA	NA	PANSS-N	rTMS	NA	10	100%	20	80,000	L-DLPFC
Garg	2016	India	20	20	31.3	N	PP	Par	83%	15%	PANSS-N	rTMS	45°	5–7	100%	10	6000	MC
Goyal	2007	India	5	5	27.4	N	ITT	Par	100%	0%	PANSS-N	rTMS	45°	10	110%	10	9800	L-PFC
Guan	2020	China	28	28	54.6	Y	ITT	Par	100%	27%	PANSS-N	rTMS	Sham coil	20	110%	40	64,000	L-DLPFC
Güleken	2020	Turkey	11	10	35.1	N	PP	Par	67%	13%	PANSS-N, SANS	rTMS	90°	20	90%	20	40,000	B-DLPFC
Hajak	2004	Germany	10	10	40.4	N	ITT	Par	40%	0%	PANSS-N	rTMS	Sham coil	10	110%	10	10,000	L-DLPFC
Holi	2004	Finland	11	11	36.0	N	ITT	Par	86%	9%	PANSS-N	rTMS	90°	10	100%	10	10,000	L-DLPFC
Huang	2016	China	19	18	39.8	N	PP	Par	100%	5%	PANSS-N	rTMS	Sham coil	10	110%	21	42,000	L-DLPFC
Klein	1999	Israel	16	15	29.7	N	PP	Par	37%	11%	PANSS-N	rTMS	90°	1	110%	10	1200	R-PFC
Kumar	2020	India	50	50	36.3	Y	ITT	Par	57%	7%	PANSS-N, SANS	rTMS	Sham coil	20	100%	20	40,000	L-DLPFC
Li	2016	Taiwan/ China	25	22	45.0	Y	ITT	Par	49%	19%	SANS, PANSS	rTMS	Sham coil	10	110%	20	30,000	L-DLPFC
Liu	2008	China	13	12	34.4	NA	NA	NA	NA	NA	PANSS-N	rTMS	NA	10	110%	20	30,000	L-DLPFC
Ma	2016	China	58	60	NA	NA	NA	NA	NA	NA	PANSS-N	rTMS	NA	10	90%	20	20,000	L-DLPFC
Mogg	2007	UK	8	9	39.1	Y	ITT	Par	94%	0%	PANSS-N	rTMS	Sham coil	10	110%	10	20,000	L-DLPFC
Novak	2006	Czech Republic	8	8	33.6	Y	PP	Par	75%	11%	PANSS-N	rTMS	90°	20	90%	10	20,000	L-DLPFC
Paillère-Martinot	2016	France	15	12	31.5	N	ITT	Par	56%	4%	SANS	rTMS	Sham coil	1	100%	10	12,000	L-STG, L-MTG
Pan	2021	China	16	19	57.0	N	ITT	Par	68%	8%	PANSS-N	rTMS	Sham coil	10	110%	20	24,000	L-DLPFC
Prikryl	2007	Czech Republic	11	11	34.1	Y	NA	Par	100%	NA	PANSS-N, SANS	rTMS	90°	10	110%	15	22,500	L-DLPFC
Prikryl	2012	Czech Republic	19	11	33.0	Y	NA	Par	100%	NA	PANSS-N	rTMS	Sham coil	10	110%	15	22,500	L-DLPFC
Prikryl	2013	Czech Republic	23	17	33.1	Y	PP	Par	100%	11%	SANS	rTMS	Sham coil	10	110%	15	30,000	L-DLPFC
Prikryl	2014	Czech Republic	18	17	33.2	N	PP	Par	100%	13%	PANSS-N	rTMS	Sham coil	10	110%	15	42,000	L-DLPFC
Quan	2015	China	78	39	46.6	Y	ITT	Par	62%	0%	PANSS-N, SANS	rTMS	90°	10	80%	20	16,000	L-DLPFC
Rabany	2014	Israel	15	8	34.8	Y	ITT	Par	70%	17%	PANSS-N, SANS	deep-TMS	NA	20	120%	20	19,200	L-DLPFC
Ren	2011	China	12	11	34.2	NA	NA	NA	NA	NA	PANSS-N	rTMS	NA	20	80%	10	8000	B-DLPFC
Rosa	2007	Brazil	6	5	31.9	N	NA	Par	55%	0%	PANSS-N	rTMS	Sham coil	1	90%	10	9600	L-TPC
Rosenberg	2012	Israel	5	5	39.2	N	PP	Par	78%	44%	SANS	deep-TMS	Sham coil	1	110%	10	6000	L-TPC
Saba	2006	France	8	8	30.6	N	PP	Par	81%	11%	PANSS-N	rTMS	Sham coil	1	80%	14	4200	L-TPC
Schneider	2008	USA	33	15	41.1	Y	PP	Par	33%	6%	SANS	rTMS	Sham coil	1-10	110%	20	Var	L-DLPFC
Singh	2020	India	15	15	31.0	Y	ITT	Par	57%	13%	PANSS-N, SANS	rTMS	Sham coil	20	100%	20	40,000	L-DLPFC
Tikka	2017	India	8	7	26.5	N	PP	Par	NA	25%	PANSS-N	cTBS	Sham coil	50	80%	10	9000	R-IPL
Wang	2015	China	41	42	42.3	NA	NA	NA	NA	NA	PANSS-N	rTMS	NA	10	110%	20	48,000	L-DLPFC
Wang	2020	China	25	25	NA	N	NA	Par	NA	NA	PANSS-N, SANS	iTBS	NA	NA	NA	14	NA	L-DLPFC
Wen-Xiang	2012	China	76	31	42.7	N	ITT	Par	71%	12%	PANSS-N	rTMS	Sham coil	1	80%	20	16,000	L-DLPFC
Wobrock	2015	Germany	76	81	35.3	Y	ITT	Par	75%	10%	PANSS-N	rTMS	45°	10	110%	15	15,000	L-DLPFC
Xiu	2020	China	67	30	52.4	Y	ITT	Par	100%	19%	PANSS-N	rTMS	Sham coil	10–20	110%	40	Var	L-DLPFC
Xu	2006	China	33	34	38.0	NA	NA	NA	NA	NA	PANSS-N	rTMS	NA	NA	80%	10	Var	L-DLPFC, B-PC
Xu	2015	China	60	30	45.3	NA	NA	NA	NA	NA	PANSS-N	rTMS	NA	5–10	80%	10	25,000	L-DLPFC
Zhang	2010	China	15	15	38	NA	NA	NA	NA	NA	PANSS-N	TBS	NA	NA	80%	20	48,000	L-DLPFC
Zhang	2015	China	35	34	39.9	NA	NA	NA	NA	NA	PANSS-N	rTMS	NA	10	NA	20	16,000	L-DLPFC
Zhao	2014	China	71	22	47.9	Y	PP	Par	56%	3%	PANSS-N, SANS	rTMS, iTBS	180°	10–50	Var	20	Var	L-DLPFC
Zheng	2012	China	56	17	56.3	NA	NA	Par	NA	NA	PANSS-N	rTMS, TBS	NA	10–20	80%	5	2000–6000	L-DLPFC
Zhuo	2019	China	33	27	30.0	Y	PP	Par	37%	14%	PANSS-N, SANS	rTMS	180°	20	90%	20	40,000	L-DLPFC

*N*_*a*_ Number of participants active group, *N*_*c*_ Number of participants control group, *PNS* Patients with predominant negative symptoms, *N* No, *Y* Yes, *ITT* Intention-to-treat, *PP* Per protocol, *NA* Not available, *Par* Parallel, *PANSS*-*N* Positive and Negative Syndrome Scale – Negative subscale, *SANS* Scale for the Assessment of Negative Symptoms, *BPRS*-*N/D* Brief Psychiatric Rating Scale – Negative/Disorganization factor. *TMS* Transcranial Magnetic Stimulation, *Hz* Herz (frequency of stimulation), % *MT* Percent of Motor Threshold, *rTMS* repetitive Transcranial Magnetic Stimulation, *TBS* theta burst stimulation, *cTBS* continuous theta burst stimulation, *iTBS* Intermittent theta burst stimulation, *B*-*DLPFC* Bilateral dorsolateral prefrontal cortex, *L*-*DLPFC* Left dorsolateral prefrontal cortex, *R*-*DLPFC* Right dorsolateral prefrontal cortex, *MC* Medullar cerebellum, *L*-*TPC* Left tempero-parietal cortex, *L*-*STS* Left superior temporal sulcus, *B*-*PFC* Bilateral prefrontal cortex, *L*-*PFC* Left prefrontal cortex, *R*-*PFC* Right prefrontal cortex, *B*-*PC* Bilateral parietal cortex, *L*-*STG* Left superior temporal gyrus, *L*-*MTG* Left medial temporal gyrus, *R*-*IPL* Right inferior parietal lobule. ° in the sham type column indicates if the coil was rotated as sham method and by how many degrees.

The 57 studies included 2633 participants, of whom 1481 received active treatment and 1152 sham treatment. In the 55 studies (*n* = 2525) that reported the specific diagnoses of the participants, 98.9% had schizophrenia and 1.1% had schizoaffective disorder. The two remaining studies reported that the participants had either schizophrenia or schizoaffective disorder, without providing the distribution between the two. The studies were conducted in 15 different countries, of which China was the most common (*n* = 25). Almost all included studies reported the outcome using PANSS-N or SANS with only one study using the Brief Psychiatric Rating Scale – Negative/Disorganized factor (BPRS-N/D)^69^.

Several different active TMS modalities were used in the included trials, with some testing more than one active modality against sham treatment: rTMS (48 studies, 10 used ≤1 Hz, 39 used >1 Hz, 42 used unilateral treatment, and eight bilateral or midline treatment), theta burst stimulation (TBS) (9 studies, 5 used intermittent TBS (iTBS), 1 used continuous TBS (cTBS), and 3 used unspecified TBS), and deep-TMS (2 studies). The mean total number of TMS pulses per trial was 25,684 varying from 1200 to 80,000 with an average of 1455 pulses per treatment. The majority of the studies (*n* = 39) had the left dorsolateral prefrontal cortex (L-DLPFC) as the primary stimulation target.

### Risk of bias of individual studies

Eight studies were regarded as having low risk of bias, 10 studies with “some concerns”, and 23 studies with high risk of bias (Supplementary Table 1). The most common reason for “some concerns” was insufficient reporting whether the randomization sequence was concealed adequately (domain A). Improper analysis (e.g. “per protocol” analysis, domain B) and missing outcome data (domain C) were the most common reasons (*n* = 12 and *n* = 21, respectively, with *n* = 11 having both) for a study being regarded as having high risk of bias.

### Results of individual studies

**Fig. 2:**
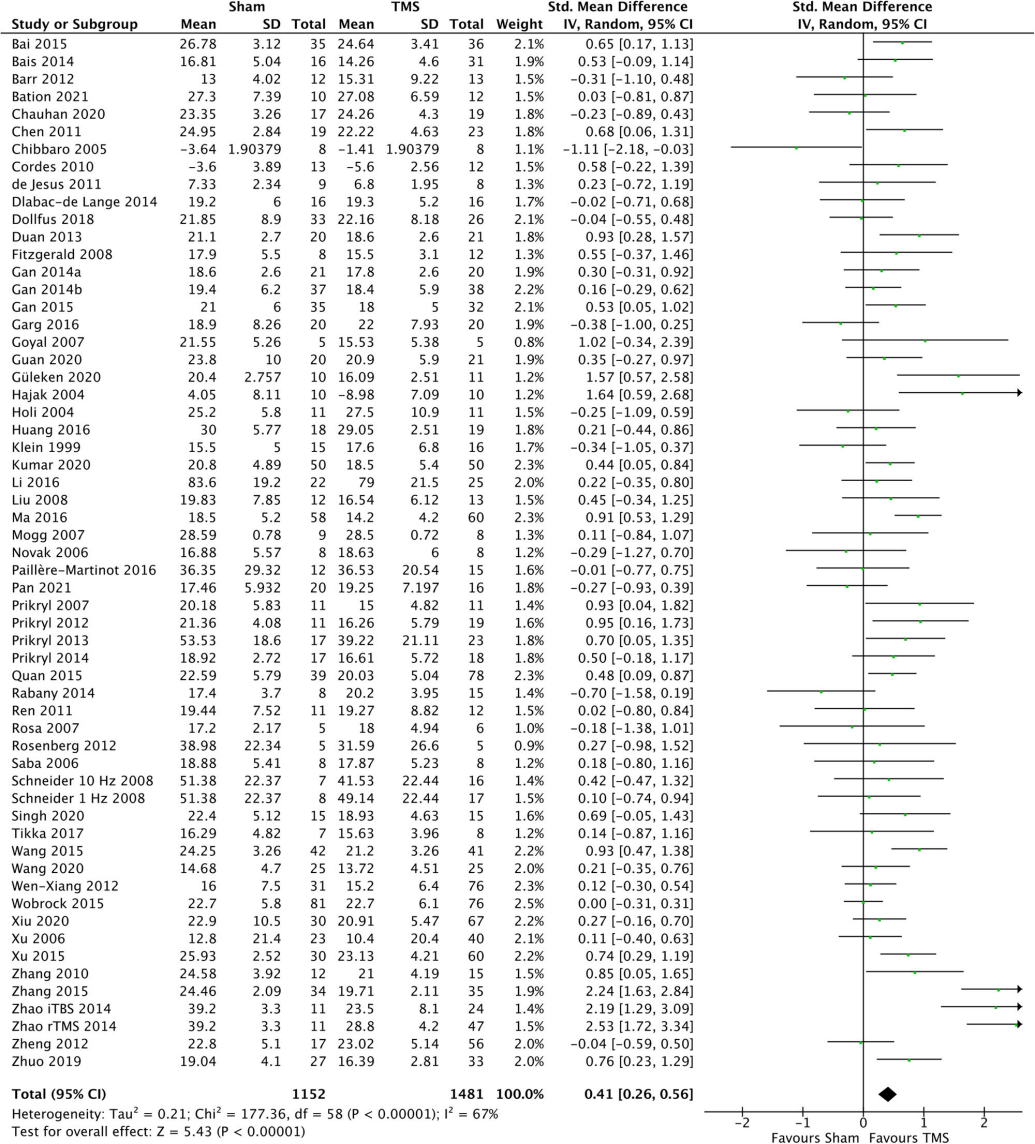
Forest plot of standardized mean differences (effect size) of TMS on negative symptoms.

Standardized mean differences for the included studies are shown in Fig. 2. Eighteen studies showed a statistically significant superior effect of TMS compared to sham treatment^21,22,38,78,79,81,82,88–90,95,96,100–103,105,106^ and one study found a statistically significant superior effect of sham treatment^36^. The remaining studies did not show a statistically significant difference between the treatment groups. There was considerable heterogeneity between the included studies (*I*^2^ = 67%).

### Synthesis of results

**Fig. 3:**
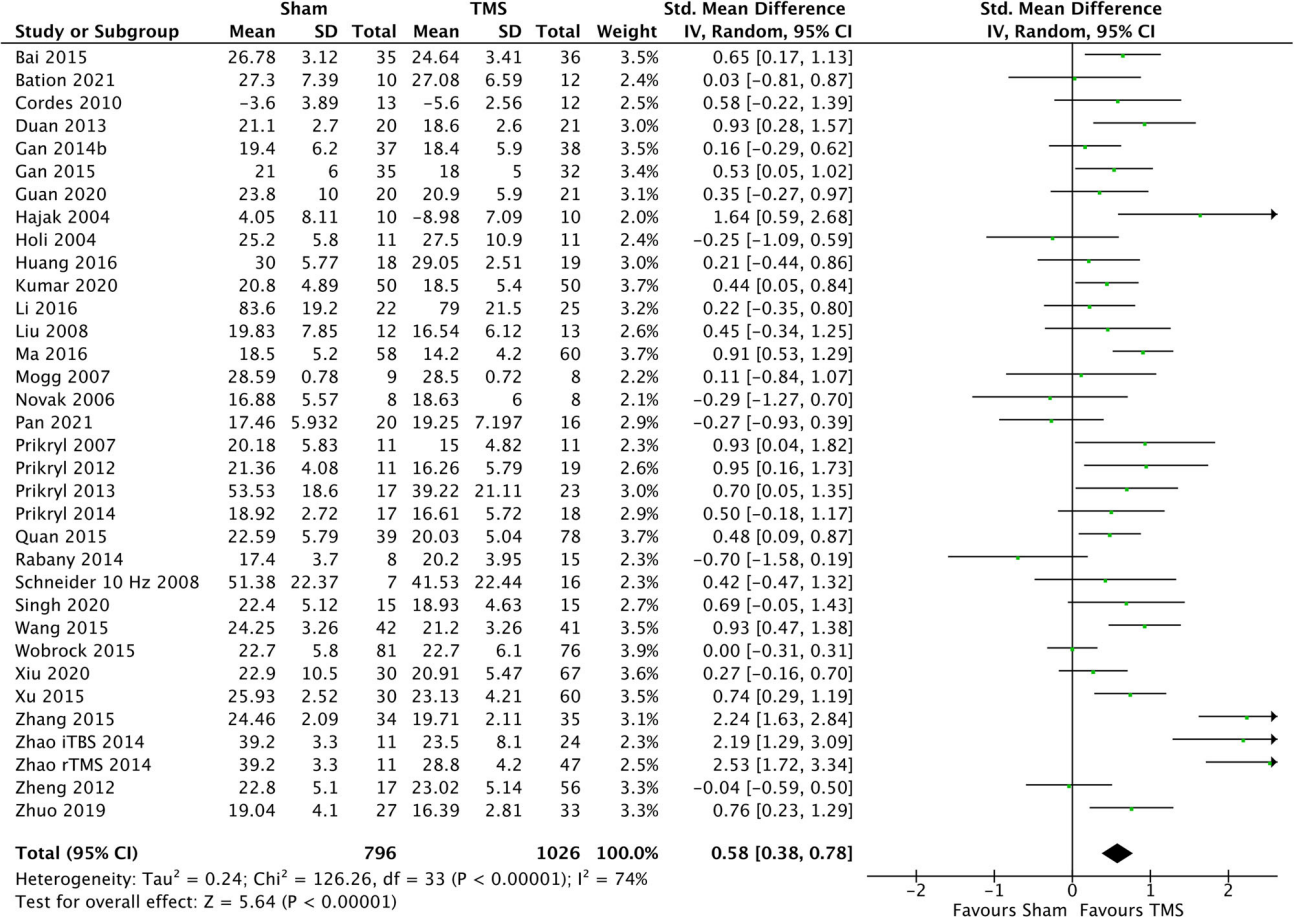
Forest plot of standardized mean differences (effect sizes) of TMS targeting negative symptoms via stimulation of the left dorsolateral prefrontal cortex (L-DLPFC) at  > 1 Hz.

As evident from the forest plot in Fig. 2, the overall SMD was 0.41 (95%CI: 0.26; 0.56, *p* < 0.001) in favour of TMS, corresponding to a NNT of 5. The results of the subgroup analyses are available in Table 2A and the results of sensitivity analyses I–IV in Table 2B. Using follow-up data from at least four weeks after end of treatment (see Supplementary Table 2 for further details) yielded an SMD of 0.27 (95%CI: 0.05; 0.49). The effect in participants with predominantly negative symptoms was substantial (SMD = 0.50, 95%CI: 0.25; 0.74), while no effect on depressive symptoms was observed (SMD = 0.02, 95%CI: −0.17; 0.20) (see Supplementary Table 3). Stimulation of the L-DLPFC had a statistically significant (*p* = 0.0002) larger effect than other sites (SMD = 0.55, 95%CI: 0.38; 0.72 vs. SMD = 0.04, 95%CI: −0.18; 0.25), however, there was considerable methodological heterogeneity in the “other sites” category. The SMD of different types of TMS did not statistically significantly differ (*p* = 0.28) (SMD = 0.49, 95%CI: 0.03; 0.95 for TBS, SMD = 0.43, 95%CI: 0.27; 0.59 for rTMS and SMD = −0.32, 95%CI: −1.24; 0.61 for deep-TMS). For forest plots of these subgroup analyses, see Supplementary Figs. 1–5. The sensitivity analyses following exclusion of (I) studies with data taken from graphs (SMD = 0.43, 95%CI: 0.27; 0.59), (II) studies with a high risk of bias (SMD = 0.42, 95%CI: 0.14; 0.73), (III) studies using change-from-baseline scores (SMD = 0.43, 95%CI: 0.28; 0.57), or (IV) studies with data extracted from other reviews (SMD = 0.32, 95%CI: 0.15; 0.49), did not impact the effect estimate substantially. A separate forest plot of the standardized mean differences (effect sizes) of the TMS studies targeting the left dorsolateral prefrontal cortex (L-DLPFC) at >1 Hz is shown in Fig. 3.

**Table 2:** Effect sizes of TMS on negative symptoms.

A. Overall effect size of TMS on negative symptoms and results from the subgroup analyses.						
		*N*	SMD (95% confidence interval)	*p*	*I* ^2^	*p* difference between groups
**Analyses**						
Overall standardized mean difference (SMD)		2633	0.41 (0.26, 0.56)	**<0.00001**	67%	
Follow-up (≥4 weeks after end of treatment		695	0.27 (0.05, 0.49)	**0.02**	42%	
Participants with PNS		1037	0.50 (0.25, 0.74)	**0.0001**	68%	
Depressive symptoms		743	0.02 (−0.17, 0.20)	0.86	30%	
**Stratified analyses**						
Site	L-DLPFC	2101	0.55 (0.38, 0.72)	**<0.00001**	70%	**0.0002**
	Other	532	0.04 (−0.18, 0.25)	0.74	27%	
Type	rTMS	2259	0.43 (0.27, 0.59)	**<0.00001**	68%	0.28
	TBS	268	0.49 (0.03, 0.95)	**0.04**	69%	
	Deep-TMS	33	−0.32 (−1.24, 0.61)	0.50	35%	
Stimulation Frequency (rTMS only)	1 Hz	297	0.05 (−0.21, 0.30)	0.72	5%	**0.003**
	> 1 Hz	1972	0.51 (0.33, 0.59)	**<0.00001**	70%	
Stimulation intensity	<100% of MT	1088	0.33 (0.11, 0.55)	**0.003**	63%	0.89
	≥100% of MT	1368	0.35 (0.20, 0.50)	**<0.00001**	40%	
Age^a^	≤median	1026	0.34 (0.17, 0.51)	**0.0001**	41%	0.43
	>median	1439	0.46 (0.22, 0.70)	**0.0002**	78%	

B. Results from the sensitivity analyses.						
Analyses		*N*	SMD (95% confidence interval)	*p*	*I* ^2^	
Excluding data from graphs		2451	0.43 (0.27, 0.59)	<0.00001	69%	
Excluding high risk of Bias studies^b^		751	0.43 (0.14, 0.73)	0.004	71%	
Excluding change-from-baseline studies		2592	0.43 (0.28, 0.57)	<0.00001	67%	
Excluding data taken from earlier reviews		1725	0.32 (0.15, 0.49)	0.0002	61%	

Bold values indicate statistical significance.

*L-DLPFC* left dorsolateral prefrontal cortex, *rTMS* repetitive transcranial magnetic stimulation, *MT* motor threshold, *TBS* theta burst stimulation, *deep-TMS* deep transcranial magnetic stimulation, *PNS* predominant negative symptoms.

^a^Median = 35.5 years.

^b^Non-English language articles also excluded as they were not evaluated. Some studies had several active treatment groups and are included in several analyses.

### Risk of bias across studies

**Fig. 4:**
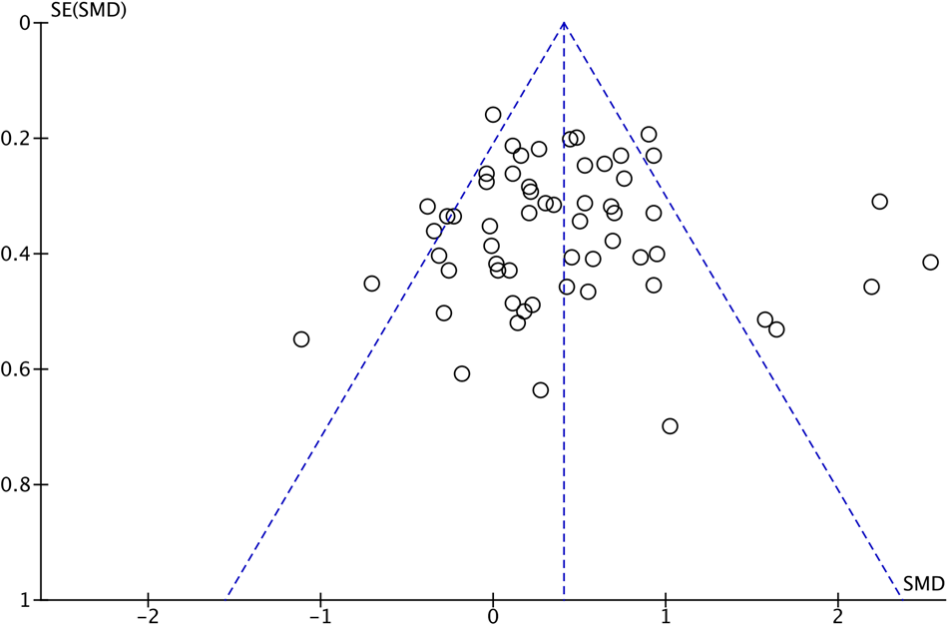
Funnel plot examining study precision versus effect size. SE Standard error, SMD Standardized mean difference.

Based on the funnel plot examining study precision versus effect size (Fig. 4), we saw no qualitative evidence of asymmetry and this was confirmed with Egger’s regression (*p* = 0.9498). There were, however, outlying studies on both sides of the confidence interval. Notably in this regard, the sensitivity analysis (no. V) involving stepwise exclusion of the 10 most outlying studies yielded no substantial change in the efficacy estimate (see Supplementary Table 4).

## Discussion

Based on meta-analysis of 57 studies with a total of 2633 participants with schizophrenia (the vast majority) or schizoaffective disorder, we found a superior effect of active TMS on negative symptoms compared to sham treatment. The SMD was 0.41 (95%CI: 0.26; 0.56) in favour of active TMS, translating to a NNT of 5. This result aligns with those from prior meta-analyses on the subject, as Aleman et al.^15^ found an SMD of 0.64 (95%CI: 0.32; 0.96), He et al.^16^ an SMD of 0.41 (95%CI: −0.35; 1.16), Wang et al.^18^ an SMD of 0.40 (95%CI: 0.18; 0.62), and Osoegawa et al.^17^ an SMD of 0.19 (95%CI: 0.07; 0.32). The superiority of active TMS on negative symptoms remained statistically significant in sensitivity analyses following (a) exclusion of data extracted from graphs, (b) exclusion of studies deemed to be at high risk of bias, and (c) exclusion of studies reporting change-from-baseline scores, respectively. Subgroup analyses suggested that using >1 Hz stimulation (SMD = 0.51 vs. SMD = 0.05, *p* = 0.003) and targeting the L-DLPFC (SMD = 0.55 vs. SMD = 0.04, *p* = 0.0002) may be more effective. However, there was considerable heterogeneity across the included studies and these results should therefore be considered tentative. In contrast to the meta-analysis by Aleman et al.^15^, we found no support for the suggestion that TMS should have a particularly beneficial effect upon negative symptoms among younger patients (SMD = 0.34 vs. SMD = 0.46, *p* = 0.43). The meta-analysis by Aleman et al. was, however, based on data from a much smaller number of studies/participating patients compared to the present work. Indeed, while there are prior meta-analysis on the effect of TMS on negative symptoms, our updated version covers substantially more studies and participants (138% more studies and 219% more participants than Aleman et al.^15^, 714% more studies and 539% more participants than He et al.^16^, 97% more studies and 83% more participants than Wang et al.^18^, and 138% more studies and 142% more participants than Osoegawa et al.^17^) and should therefore be more representative of the state-of-the-art.

Several different brain areas were targeted by TMS in the studies included in this synthesis, in which subgroup analyses suggested that stimulation of the L-DLPFC may be particularly beneficial (SMD = 0.55). These results align with earlier studies that have found an inverse correlation between frontal lobe size and glucose metabolism, and negative symptom severity^107,108^. Together with small sample size, this variability in target could in part explain why only eighteen studies found statistically significant effects of TMS since 17 of these targeted L-DLPFC. Hence, increased activity in the L-DLPFC due to magnetic stimulation could be the mechanism of action underlying the effect on negative symptoms of TMS as proposed in several of the largest included studies^25,87,88^. Moreover, there is an increasing body of data suggesting that the DLPFC has a privileged relationship with other structures implicated in negative symptoms, including the midline cerebellum^109^. For these reasons, circuitries involving the DLPFC will likely receive substantial attention in future efforts to relieve negative symptoms of schizophrenia and related psychotic disorders. Conversely, TMS of “other targets” than the L-DLPFC yielded no positive effect compared to sham treatment (SMD = 0.06). While there is considerable methodological heterogeneity among these studies, the quantitative synthesis converged on a null effect with low statistical heterogeneity. Furthermore, several of the studies targeting other sites than the L-DLPFC had other primary endpoints such as the severity of auditory hallucinations (the temporo-parietal cortex as target) with negative symptom severity as a secondary outcome^36,40,67,71,77,84^, which likely contributes to the lack of effect compared to sham treatment.

There are limitations to this study, which should be acknowledged by the readers. First, as there are phenomenological overlaps between negative and depressive symptoms and since depression responds well to TMS^110,111^, the relief of depressive symptoms during treatment could potentially confound the estimation of the effect on negative symptoms. However, the results from our analysis of data from studies measuring depressive symptoms in the context of schizophrenia (no effect of TMS) do not support this explanation. Second, 56% of the evaluated studies were regarded as having “high risk of bias”, which is a substantially larger proportion compared to the 13% reported in the review by Wang et al.^18^. This difference, however, is predominantly a consequence of classification as we used the Cochrane Risk of Bias Tool 2.0, while Wang et al. used the Cochrane Risk of Bias Tool 1.0. The most common reason for studies being considered as “high risk” in the context of the present review was missing data. We employed a relatively conservative 10% cut-off for missing data, but there is no agreed upon threshold (https://training.cochrane.org/handbook/current/chapter-08#section-8-5) and the proportion of studies classified as “high risk of bias” can thus vary considerably between reviews. Also, publication bias seems unlikely as Egger’s regression test showed non-significant results (*p* = 0.9498), but cannot be ruled out. The two most outlying studies^88,103^ did not differ substantially in their treatment parameters as both targeted the L-DLPFC with >1 Hz over 20 days of treatment, however, the stimulation intensity (percent of MT) was not described for Zhang 2015. Third, we used a broad search strategy, but relevant studies may have been missed nevertheless. However, assuming that such potential misses occur at random, it should not have affected the reported efficacy estimates. Fourth, the inclusion of data drawn from reviews and graphs is suboptimal. However, the analyses excluding these data yielded results equivalent to those from the primary analysis. Fifth, there was significant heterogeneity in outcome across the included studies, with *I*^2^-assessments at 50% or above in all but seven cases (66% in the primary analysis; Fig. 2). While this is likely partly due to the considerable heterogeneity of the TMS treatment provided across the included studies, other sources of heterogeneity, such as differences in sham conditions, patient populations, outcome measures, or random chance, are also likely contributors. Relatedly, in the review by He et al.^16^, a univariate meta-regression of stimulation frequency, total simulation, motor threshold, stimulation site, study design, and type of coil was conducted. None of these factors were shown to be the main source of heterogeneity. Seventh, 24 studies could not be included due to unavailable data. These studies were, however, generally smaller, which reduces the impact of this limitation.

In conclusion, this systematic review and meta-analysis of data from sham-controlled studies suggests that TMS is efficacious in the treatment of negative symptoms of schizophrenia. Although it appears that targeting the L-DLPFC and using a stimulation frequency >1 Hz are the most efficacious settings, the optimal treatment parameters are yet to be established.

## Supplementary information

Supplementary Material

## Data Availability

All data are available in the manuscript, figures, tables, and supplementary files.
