# Supplementary material for: The efficacy of transcranial magnetic stimulation (TMS) for negative symptoms in schizophrenia: a systematic review and meta-analysis
Source: Schizophrenia (Heidelb). 2022 Apr 9;8(1):35. doi: 10.1038/s41537-022-00248-6 (PMC9261093; doi:10.1038/s41537-022-00248-6)
Supplement: Supplementary file 1 — Supplementary Material [file 41537_2022_248_MOESM1_ESM.pdf]

Supplementary Material

The efficacy of transcranial magnetic stimulation (TMS) for negative symptoms in schizophrenia: A systematic review and meta-analysis

Rasmus Lorentzen[1,2], Tuan D. Nguyen[1,2], Alexander McGirr[3,4,5],
Fredrik Hieronymus[1,2,6], Søren D. Østergaard[1,2]

[1]Department of Affective Disorders, Aarhus University Hospital – Psychiatry, Aarhus, Denmark
[2]Department of Clinical Medicine, Aarhus University, Aarhus, Denmark
[3]Hotchkiss Brain Institute, University of Calgary, Calgary, Canada
[4]Department of Psychiatry, Cumming School of Medicine, University of Calgary, Calgary, Canada
[5]Mathison Centre for Mental Health Research and Education, University of Calgary, Calgary, Canada
[6]Department of Pharmacology, Sahlgrenska Academy, University of Gothenburg, Gothenburg, Sweden

Search strategy

PubMed:
("schizophreni*" OR "schizoaffective disorder" OR "schizophreniform disorder" OR "schizophrenia"[MeSH Terms] OR "negative symptom*" OR "CHR" OR "Clinical High Risk" OR "Ultra High Risk" OR "UHR" OR "Psychotic Disorders"[MeSH Terms] OR "Psychotic Disorder*") AND ("transcranial magnetic stimulation" OR "TMS" OR "rTMS" OR "theta burst" OR "iTBS" OR "cTBS" OR "transcranial Magnetic Stimulation*"[MeSH Terms])

751 hits

EMBASE:
('schizophreni*':ab,kw,ti OR 'schizoaffective disorder':ab,ti,kw OR 'schizophreniform disorder':ab,kw,ti OR 'schizophrenia spectrum disorder'/exp OR 'psychotic disorder*':ab,kw,ti OR 'negative symptom*':ab,kw,ti OR 'CHR':ab,kw,ti OR 'Clinical High Risk':ab,kw,ti OR 'Ultra High Risk':ab,kw,ti OR 'UHR':ab,kw,ti) AND ('transcranial magnetic stimulation':ab,kw,ti OR 'tms':ab,kw,ti OR 'rtms':ab,kw,ti OR 'theta burst':ab,kw,ti OR 'itbs':ab,kw,ti OR 'cTBS':ab,kw,ti OR 'transcranial magnetic stimulation'/exp) AND ('article'/it OR 'article in press'/it OR 'review'/it)

902 hits

PsycINFO:
(schizophreni* OR "schizoaffective disorder" OR "schizophreniform disorder" OR "Psychotic Disorder*" OR "negative symptom*" OR "CHR" OR "Clinical High Risk" OR "Ultra High Risk" OR "UHR") AND ("transcranial magnetic stimulation" OR "TMS" OR "rTMS" OR "theta burst" OR "iTBS" OR "cTBS")
Source type: Scholarly Journals

699 hits

Web of Sciences (Web of Science, Core Database Collection):
(schizophreni* OR "schizoaffective disorder" OR "schizophreniform disorder" OR "Psychotic Disorder*" OR "negative symptom*" OR "CHR" OR "Clinical High Risk" OR "Ultra High Risk" OR "UHR") AND ("transcranial magnetic stimulation" OR "TMS" OR "rTMS" OR "theta burst" OR "iTBS" OR "cTBS")
Search type: "Topic". Document type: Article, review, proceedings paper, early acces

935 hits

In total 3287 hits

After removal of **1573** duplicates: **1714 hits**

Supplementary Table 1. Risk of bias in individual studies

	Cochrane Risk of Bias 2.0 domains	Overall score

Author	Year	A	B	C	D	E	
Bais	2014	L	L	L	L	L	**L**
Barr	2012	SC	H	H	L	L	**H**
Bation	2021	L	L	L	L	L	**L**
Chauhan	2020	L	SC	L	L	L	**SC**
Chibbaro	2005	SC	SC	H	L	L	**H**
Cordes	2010	SC	H	L	L	L	**H**
de Jesus	2011	L	L	L	L	L	**L**
Dlabac-de Lange	2014	L	L	L	L	L	**L**
Dollfus	2018	SC	H	H	L	L	**H**
Fitzgerald	2008	L	SC	H	L	L	**H**
Garg	2016	SC	H	H	L	L	**H**
Goyal	2007	H	L	L	L	L	**H**
Guan	2020	L	SC	H	L	L	**H**
Güleken	2020	H	SC	H	L	L	**H**
Hajak	2004	SC	L	L	L	L	**SC**
Holi	2004	L	L	L	L	L	**L**
Huang	2016	SC	SC	L	L	L	**SC**
Klein	1999	SC	H	H	L	L	**H**
Kumar	2020	L	L	L	L	L	**L**
Li	2016	SC	SC	L	L	L	**SC**
Mogg	2007	L	L	L	L	L	**L**
Novak	2006	SC	H	H	L	L	**H**
Paillère-Martinot	2016	L	L	L	L	L	**L**
Pan	2021	SC	L	L	L	L	**SC**
Prikryl	2007	SC	L	L	L	L	**SC**
Prikryl	2012	SC	H	H	L	L	**H**
Prikryl	2013	SC	H	H	L	L	**H**
Prikryl	2014	SC	H	H	L	L	**H**
Quan	2015	SC	SC	L	L	L	**SC**
Rabany	2014	SC	L	H	L	L	**H**
Rosa	2007	SC	L	L	L	L	**SC**
Rosenberg	2012	L	H	H	L	L	**H**
Saba	2006	SC	SC	H	L	L	**H**
Schneider	2008	SC	SC	L	L	L	**SC**
Singh	2020	L	L	H	L	L	**H**
Tikka	2017	L	H	H	L	L	**H**
Wang	2020	SC	SC	H	L	L	**H**
Wobrock	2015	L	SC	H	L	L	**H**
Xiu	2020	L	SC	H	L	L	**H**
Zhao	2014	SC	SC	L	L	L	**SC**
Zhuo	2019	SC	H	H	L	L	**H**

A: Randomization process, B: Deviations from intended interventions, C: Missing outcome data, D: Measurement of the outcome, E: Selection of the reported result, L: Low risk, SC: Some concerns, H: High risk.

Supplementary Table 2. Studies with follow up data from at least four weeks after end of treatment

Author	Year	Follow up time	Follow up score (SD)$_{active}$		Follow up score (SD)$_{control}$	
Bation	2021	6 months	22.83	(6.043)	27.3	(10.38)
Chibbaro	2005	8 weeks	-8.28	(2.72)	0.45	(0.19)
de Jesus	2011	4 weeks	9.5	(3.81)	12.56	(4.79)
Dlabac-de Lange	2014	3 months	18.1	(4.6)	18.2	(6)
Dollfus	2018	4 weeks	22.77	(7.81)	22.97	(8.39)
Li	2016	4 months	15.7	(4.93)	19.3	(5.92)
Liu	2008	4 weeks	72.5	(16.8)	83.5	(20.5)
Novak	2006	6 weeks	18	(5.73)	15.38	(5.1)
Rabany	2014	4 weeks	18.6	(4.93)	15	(5.83)
Rosa	2007	4 weeks	18	(4.8)	18.75	(0.5)
Schneider	2008	4 weeks	46.59	(22.55)	50.93	(22.37)
Wang	2020	2 months	12.08	(3.66)	14.52	(4.78)
Wobrock	2015	84 days	19.99	(50.82)	20.32	(63)
Xiu	2020	6 months	20.76	(8.48)	22.2	(7.9)

SD: Standard deviation

Supplementary Table 3. Studies with data on depressive symptoms

Author	Year	Depression scale used	Post treatment depression score (SD)$_{active}$		Post treatment depression score (SD)$_{control}$	
Barr	2012	CDSS	2.23	(1.87)	1.58	(2.5)
de Jesus	2011	BPRS-DF	3.37	(2.26)	3.55	(3.24)
Dlabac-de Lange	2014	MADRS	16.3	(7.8)	11.8	(6.8)
Fitzgerald	2008	CDSS	7.2	(5.9)	3.5	(3.8)
Garg	2016	CDSS	6.3	(3.45)	6.5	(3.68)
Goyal	2007	CDSS	0	(0)	0.8	(0.836)
Guan	2020	PANSS-DF	3.9	(2)	4.2	(1.6)
Huang	2016	MADRS	14.89	(5.52)	12.39	(2.57)
Klein	1999	HDRS	8.6	(3.5)	6.9	(4)
Kumar	2020	CDSS	0.12	(0.44)	0.12	(0.72)
Mogg	2007	HADS-D	2.5	(3.1)	5.2	(3.3)
Prikryl	2007	CDSS	0	(0)	0.73	(1.19)
Prikryl	2013	CDSS	0.04	(0.21)	0.76	(1.48)
Prikryl	2014	CDSS	0.92	(0.78)	1.09	(1.51)
Rabany	2014	CDSS	5.8	(3.23)	5.5	(4.78)
Singh	2020	CDSS	1.33	(0.97)	1.33	(1.23)
Wang	2020	HDRS	3.76	(1.45)	5.4	(3:33)
Wobrock	2015	CDSS	4.4	(3.5)	4.6	(4.4)

CDSS: Calgary Depression Scale for Schizophrenia, BPRS-DF: Brief Psychiatric Rating Scale – depressive factor, MADRS: Montgomery-Asberg Depression Rating Scale, PANSS-DF: Positive and Negative Syndrome Scale – Depressive Factor, HDRS: Hamilton Depression Rating Scale, HADS-D: Hospital Anxiety and Depression Score – Depression score.

Supplementary Table 4. Stepwise exclusion of the ten most outlying studies compared to the overall efficacy estimate (beginning with the most outlying)

**Original estimate**	**0.41 (0.26; 0.56)**	**$p < 0.00001$**
**Excluding Zhao rTMS 2014**	0.38 (0.24; 0.52)	$p < 0.00001$
**+ excluding Zhang 2015**	0.34 (0.22; 0.47)	$p < 0.00001$
**+ excluding Zhao iTBS 2014**	0.32 (0.21; 0.44)	$p < 0.00001$
**+ excluding Chibarro 2005**	0.34 (0.22; 0.45)	$p < 0.00001$
**+ excluding Hajak 2004**	0.33 (0.21; 0.44)	$p < 0.00001$
**+ excluding Güleken 2020**	0.31 (0.21; 0.42)	$p < 0.00001$
**+ excluding Rabany 2014**	0.33 (0.22; 0.43)	$p < 0.00001$
**+ excluding Garg 2016**	0.34 (0.24; 0.45)	$p < 0.00001$
**+ excluding Klein 1999**	0.36 (0.25; 0.46)	$p < 0.00001$
**+ excluding Barr 2012**	0.36 (0.26; 0.47)	$p < 0.00001$

Supplementary Figure 1. Subgroup analysis stratified by targeted brain structure (Left dorsolateral prefrontal cortex (L-DLPFC) or "other site"

	Sham			TMS				Std. Mean Difference
**Study or Subgroup**	**Mean**	**SD**	**Total**	**Mean**	**SD**	**Total**	**Weight**	**IV, Random, 95% CI**
**1.2.1 L-DLPFC**								
Bai 2015	26.78	3.12	35	24.64	3.41	36	2.1%	0.65 [0.17, 1.13]
Bation 2021	27.3	7.39	10	27.08	6.59	12	1.5%	0.03 [−0.81, 0.87]
Chen 2011	24.95	2.84	19	22.22	4.63	23	1.8%	0.68 [0.06, 1.31]
Cordes 2010	−3.6	3.89	13	−5.6	2.56	12	1.5%	0.58 [−0.22, 1.39]
Duan 2013	21.1	2.7	20	18.6	2.6	21	1.8%	0.93 [0.28, 1.57]
Gan 2014a	18.6	2.6	21	17.8	2.6	20	1.9%	0.30 [−0.31, 0.92]
Gan 2014b	19.4	6.2	37	18.4	5.9	38	2.2%	0.16 [−0.29, 0.62]
Gan 2015	21	6	35	18	5	32	2.1%	0.53 [0.05, 1.02]
Guan 2020	23.8	10	20	20.9	5.9	21	1.9%	0.35 [−0.27, 0.97]
Hajak 2004	4.05	8.11	10	−8.98	7.09	10	1.2%	1.64 [0.59, 2.68]
Holi 2004	25.2	5.8	11	27.5	10.9	11	1.5%	−0.25 [−1.09, 0.59]
Huang 2016	30	5.77	18	29.05	2.51	19	1.8%	0.21 [−0.44, 0.86]
Kumar 2020	20.8	4.89	50	18.5	5.4	50	2.3%	0.44 [0.05, 0.84]
Li 2016	83.6	19.2	22	79	21.5	25	2.0%	0.22 [−0.35, 0.80]
Liu 2008	19.83	7.85	12	16.54	6.12	13	1.5%	0.45 [−0.34, 1.25]
Ma 2016	18.5	5.2	58	14.2	4.2	60	2.3%	0.91 [0.53, 1.29]
Mogg 2007	28.59	0.78	9	28.5	0.72	8	1.3%	0.11 [−0.84, 1.07]
Novak 2006	16.88	5.57	8	18.63	6	8	1.2%	−0.29 [−1.27, 0.70]
Prikryl 2007	20.18	5.83	11	15	4.82	11	1.4%	0.93 [0.04, 1.82]
Prikryl 2012	21.36	4.08	11	16.26	5.79	19	1.6%	0.95 [0.16, 1.73]
Prikryl 2013	53.53	18.6	17	39.22	21.11	23	1.8%	0.70 [0.05, 1.35]
Prikryl 2014	18.92	2.72	17	16.61	5.72	18	1.8%	0.50 [−0.18, 1.17]
Quan 2015	22.59	5.79	39	20.03	5.04	78	2.3%	0.48 [0.09, 0.87]
Rabany 2014	17.4	3.7	8	20.2	3.95	15	1.4%	−0.70 [−1.58, 0.19]
Ren 2011	19.44	7.52	11	19.27	8.82	12	1.5%	0.02 [−0.80, 0.84]
Schneider 10 Hz 2008	51.38	22.37	7	41.53	22.44	16	1.4%	0.42 [−0.47, 1.32]
Schneider 1 Hz 2008	51.38	22.37	8	49.14	22.44	17	1.5%	0.10 [−0.74, 0.94]
Singh 2020	22.4	5.12	15	18.93	4.63	15	1.6%	0.69 [−0.05, 1.43]
Wang 2015	24.25	3.26	42	21.2	3.26	41	2.2%	0.93 [0.47, 1.38]
Wang 2020	14.68	4.7	25	13.72	4.51	25	2.0%	0.21 [−0.35, 0.76]
Wen−Xiang 2012	16	7.5	31	15.2	6.4	76	2.3%	0.12 [−0.30, 0.54]
Wobrock 2015	22.7	5.8	81	22.7	6.1	76	2.5%	0.00 [−0.31, 0.31]
Xiu 2020	22.9	10.5	30	20.91	5.47	67	2.2%	0.27 [−0.16, 0.70]
Xu 2015	25.93	2.52	30	23.13	4.21	60	2.2%	0.74 [0.29, 1.19]
Zhang 2010	24.58	3.92	12	21	4.19	15	1.5%	0.85 [0.05, 1.65]
Zhang 2015	24.46	2.09	34	19.71	2.11	35	1.9%	2.24 [1.63, 2.84]
Zhao iTBS 2014	39.2	3.3	11	23.5	8.1	24	1.4%	2.19 [1.29, 3.09]
Zhao rTMS 2014	39.2	3.3	11	28.8	4.2	47	1.5%	2.53 [1.72, 3.34]
Zheng 2012	22.8	5.1	17	23.02	5.14	56	2.0%	−0.04 [−0.59, 0.50]
Zhuo 2019	19.04	4.1	27	16.39	2.81	33	2.0%	0.76 [0.23, 1.29]
**Subtotal (95% CI)**			**903**			**1198**	**71.8%**	**0.55 [0.38, 0.72]**
Heterogeneity: Tau² = 0.20; Chi² = 129.38, df = 39 (P < 0.00001); I² = 70%								
Test for overall effect: Z = 6.23 (P < 0.00001)								
**1.2.2 Other site**								
Bais 2014	16.81	5.04	16	14.26	4.6	31	1.9%	0.53 [−0.09, 1.14]
Barr 2012	13	4.02	12	15.31	9.22	13	1.5%	−0.31 [−1.10, 0.48]
Chauhan 2020	23.35	3.26	17	24.26	4.3	19	1.8%	−0.23 [−0.89, 0.43]
Chibbaro 2005	−3.64	1.90379	8	−1.41	1.90379	8	1.1%	−1.11 [−2.18, −0.03]
de Jesus 2011	7.33	2.34	9	6.8	1.95	8	1.3%	0.23 [−0.72, 1.19]
Dlabac−de Lange 2014	19.2	6	16	19.3	5.2	16	1.7%	−0.02 [−0.71, 0.68]
Dollfus 2018	21.85	8.9	33	22.16	8.18	26	2.1%	−0.04 [−0.55, 0.48]
Fitzgerald 2008	17.9	5.5	8	15.5	3.1	12	1.3%	0.55 [−0.37, 1.46]
Garg 2016	18.9	8.26	20	22	7.93	20	1.9%	−0.38 [−1.00, 0.25]
Goyal 2007	21.55	5.26	5	15.53	5.38	5	0.8%	1.02 [−0.34, 2.39]
Güleken 2020	20.4	2.757	10	16.09	2.51	11	1.2%	1.57 [0.57, 2.58]
Klein 1999	15.5	5	15	17.6	6.8	16	1.7%	−0.34 [−1.05, 0.37]
Paillère−Martinot 2016	36.35	29.32	12	36.53	20.54	15	1.6%	−0.01 [−0.77, 0.75]
Pan 2021	17.46	5.932	20	19.25	7.197	16	1.8%	−0.27 [−0.93, 0.39]
Rosa 2007	17.2	2.17	5	18	4.94	6	1.0%	−0.18 [−1.38, 1.01]
Rosenberg 2012	38.98	22.34	5	31.59	26.6	5	0.9%	0.27 [−0.98, 1.52]
Saba 2006	18.88	5.41	8	17.87	5.23	8	1.2%	0.18 [−0.80, 1.16]
Tikka 2017	16.29	4.82	7	15.63	3.96	8	1.2%	0.14 [−0.87, 1.16]
Xu 2006	12.8	21.4	23	10.4	20.4	40	2.1%	0.11 [−0.40, 0.63]
**Subtotal (95% CI)**			**249**			**283**	**28.2%**	**0.04 [−0.18, 0.25]**
Heterogeneity: Tau² = 0.06; Chi² = 24.65, df = 18 (P = 0.13); I² = 27%								
Test for overall effect: Z = 0.33 (P = 0.74)								
**Total (95% CI)**			**1152**			**1481**	**100.0%**	**0.41 [0.26, 0.56]**
Heterogeneity: Tau² = 0.21; Chi² = 177.36, df = 58 (P < 0.00001); I² = 67%								
Test for overall effect: Z = 5.43 (P < 0.00001)								
Test for subgroup differences: Chi² = 13.75, df = 1 (P = 0.0002), I² = 92.7%								

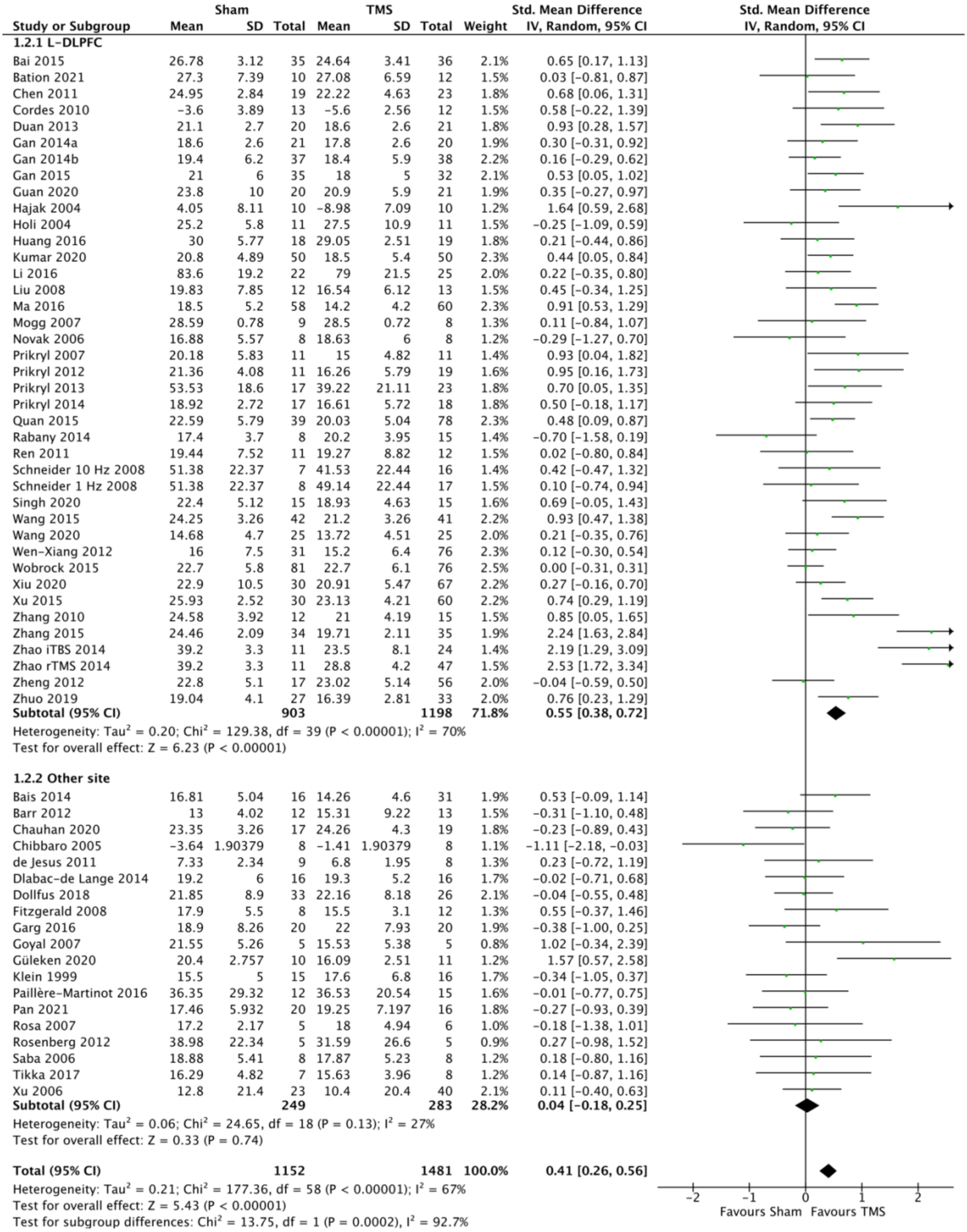

Supplementary Figure 2. Subgroup analysis stratified by type of treatment (repetitive transcranial stimulation (rTMS), theta burst stimulation (TBS), and deep transcranial stimulation (deep-TMS)

Study or Subgroup	Sham Mean	Sham SD	Sham Total	TMS Mean	TMS SD	TMS Total	Weight	Std. Mean Difference IV, Random, 95% CI
**1.2.1 rTMS**								
Bai 2015	26.78	3.12	35	24.64	3.41	36	2.2%	0.65 [0.17, 1.13]
Bais 2014	16.81	5.04	16	14.26	4.6	31	1.9%	0.53 [−0.09, 1.14]
Barr 2012	13	4.02	12	15.31	9.22	13	1.6%	−0.31 [−1.10, 0.48]
Chibbaro 2005	−3.64	1.90379	8	−1.41	1.90379	8	1.2%	−1.11 [−2.18, −0.03]
Cordes 2010	−3.6	3.89	13	−5.6	2.56	12	1.6%	0.58 [−0.22, 1.39]
de Jesus 2011	7.33	2.34	9	6.8	1.95	8	1.3%	0.23 [−0.72, 1.19]
Dlabac-de Lange 2014	19.2	6	16	19.3	5.2	16	1.8%	−0.02 [−0.71, 0.68]
Dollfus 2018	21.85	8.9	33	22.16	8.18	26	2.1%	−0.04 [−0.55, 0.48]
Duan 2013	21.1	2.7	20	18.6	2.6	21	1.8%	0.93 [0.28, 1.57]
Fitzgerald 2008	17.9	5.5	8	15.5	3.1	12	1.4%	0.55 [−0.37, 1.46]
Gan 2014b	19.4	6.2	37	18.4	5.9	38	2.2%	0.16 [−0.29, 0.62]
Gan 2015	21	6	35	18	5	32	2.2%	0.53 [0.05, 1.02]
Garg 2016	18.9	8.26	20	22	7.93	20	1.9%	−0.38 [−1.00, 0.25]
Goyal 2007	21.55	5.26	5	15.53	5.38	5	0.8%	1.02 [−0.34, 2.39]
Guan 2020	23.8	10	20	20.9	5.9	21	1.9%	0.35 [−0.27, 0.97]
Güleken 2020	20.4	2.757	10	16.09	2.51	11	1.2%	1.57 [0.57, 2.58]
Hajak 2004	4.05	8.11	10	−8.98	7.09	10	1.2%	1.64 [0.59, 2.68]
Holi 2004	25.2	5.8	11	27.5	10.9	11	1.5%	−0.25 [−1.09, 0.59]
Huang 2016	30	5.77	18	29.05	2.51	19	1.8%	0.21 [−0.44, 0.86]
Klein 1999	15.5	5	15	17.6	6.8	16	1.7%	−0.34 [−1.05, 0.37]
Kumar 2020	20.8	4.89	50	18.5	5.4	50	2.3%	0.44 [0.05, 0.84]
Li 2016	83.6	19.2	22	79	21.5	25	2.0%	0.22 [−0.35, 0.80]
Liu 2008	19.83	7.85	12	16.54	6.12	13	1.6%	0.45 [−0.34, 1.25]
Ma 2016	18.5	5.2	58	14.2	4.2	60	2.4%	0.91 [0.53, 1.29]
Mogg 2007	28.59	0.78	9	28.5	0.72	8	1.3%	0.11 [−0.84, 1.07]
Novak 2006	16.88	5.57	8	18.63	6	8	1.3%	−0.29 [−1.27, 0.70]
Paillère-Martinot 2016	36.35	29.32	12	36.53	20.54	15	1.6%	−0.01 [−0.77, 0.75]
Pan 2021	17.46	5.932	20	19.25	7.197	16	1.8%	−0.27 [−0.93, 0.39]
Prikryl 2007	20.18	5.83	11	15	4.82	11	1.4%	0.93 [0.04, 1.82]
Prikryl 2012	21.36	4.08	11	16.26	5.79	19	1.6%	0.95 [0.16, 1.73]
Prikryl 2013	53.53	18.6	17	39.22	21.11	23	1.8%	0.70 [0.05, 1.35]
Prikryl 2014	18.92	2.72	17	16.61	5.72	18	1.8%	0.50 [−0.18, 1.17]
Quan 2015	22.59	5.79	39	20.03	5.04	78	2.4%	0.48 [0.09, 0.87]
Ren 2011	19.44	7.52	11	19.27	8.82	12	1.5%	0.02 [−0.80, 0.84]
Rosa 2007	17.2	2.17	5	18	4.94	6	1.0%	−0.18 [−1.38, 1.01]
Saba 2006	18.88	5.41	8	17.87	5.23	8	1.3%	0.18 [−0.80, 1.16]
Schneider 10 Hz 2008	51.38	22.37	7	41.53	22.44	16	1.4%	0.42 [−0.47, 1.32]
Schneider 1 Hz 2008	51.38	22.37	8	49.14	22.44	17	1.5%	0.10 [−0.74, 0.94]
Singh 2020	22.4	5.12	15	18.93	4.63	15	1.7%	0.69 [−0.05, 1.43]
Wang 2015	24.25	3.26	42	21.2	3.26	41	2.2%	0.93 [0.47, 1.38]
Wen-Xiang 2012	16	7.5	31	15.2	6.4	76	2.3%	0.12 [−0.30, 0.54]
Wobrock 2015	22.7	5.8	81	22.7	6.1	76	2.5%	0.00 [−0.31, 0.31]
Xiu 2020	22.9	10.5	30	20.91	5.47	67	2.3%	0.27 [−0.16, 0.70]
Xu 2006	12.8	21.4	23	10.4	20.4	40	2.1%	0.11 [−0.40, 0.63]
Xu 2015	25.93	2.52	30	23.13	4.21	60	2.2%	0.74 [0.29, 1.19]
Zhang 2015	24.46	2.09	34	19.71	2.11	35	1.9%	2.24 [1.63, 2.84]
Zhao rTMS 2014	39.2	3.3	11	28.8	4.2	47	1.5%	2.53 [1.72, 3.34]
Zhuo 2019	19.04	4.1	27	16.39	2.81	33	2.1%	0.76 [0.23, 1.29]
**Subtotal (95% CI)**			**1000**			**1259**	**84.3%**	**0.43 [0.27, 0.59]**
Heterogeneity: Tau² = 0.20; Chi² = 146.16, df = 47 (P < 0.00001); I² = 68%								
Test for overall effect: Z = 5.22 (P < 0.00001)								
**1.2.2 TBS**								
Bation 2021	27.3	7.39	10	27.08	6.59	12	1.5%	0.03 [−0.81, 0.87]
Chauhan 2020	23.35	3.26	17	24.26	4.3	19	1.8%	−0.23 [−0.89, 0.43]
Chen 2011	24.95	2.84	19	22.22	4.63	23	1.9%	0.68 [0.06, 1.31]
Gan 2014a	18.6	2.6	21	17.8	2.6	20	1.9%	0.30 [−0.31, 0.92]
Tikka 2017	16.29	4.82	7	15.63	3.96	8	1.2%	0.14 [−0.87, 1.16]
Wang 2020	14.68	4.7	25	13.72	4.51	25	2.0%	0.21 [−0.35, 0.76]
Zhang 2010	24.58	3.92	12	21	4.19	15	1.6%	0.85 [0.05, 1.65]
Zhao iTBS 2014	39.2	3.3	11	23.5	8.1	24	1.4%	2.19 [1.29, 3.09]
**Subtotal (95% CI)**			**122**			**146**	**13.3%**	**0.49 [0.03, 0.95]**
Heterogeneity: Tau² = 0.29; Chi² = 22.34, df = 7 (P = 0.002); I² = 69%								
Test for overall effect: Z = 2.11 (P = 0.04)								
**1.2.3 deep-TMS**								
Rabany 2014	17.4	3.7	8	20.2	3.95	15	1.4%	−0.70 [−1.58, 0.19]
Rosenberg 2012	38.98	22.34	5	31.59	26.6	5	1.0%	0.27 [−0.98, 1.52]
**Subtotal (95% CI)**			**13**			**20**	**2.4%**	**−0.32 [−1.24, 0.61]**
Heterogeneity: Tau² = 0.16; Chi² = 1.54, df = 1 (P = 0.21); I² = 35%								
Test for overall effect: Z = 0.67 (P = 0.50)								
**Total (95% CI)**			**1135**			**1425**	**100.0%**	**0.42 [0.27, 0.57]**
Heterogeneity: Tau² = 0.21; Chi² = 174.68, df = 57 (P < 0.00001); I² = 67%								
Test for overall effect: Z = 5.48 (P < 0.00001)								
Test for subgroup differences: Chi² = 2.54, df = 2 (P = 0.28), I² = 21.1%								

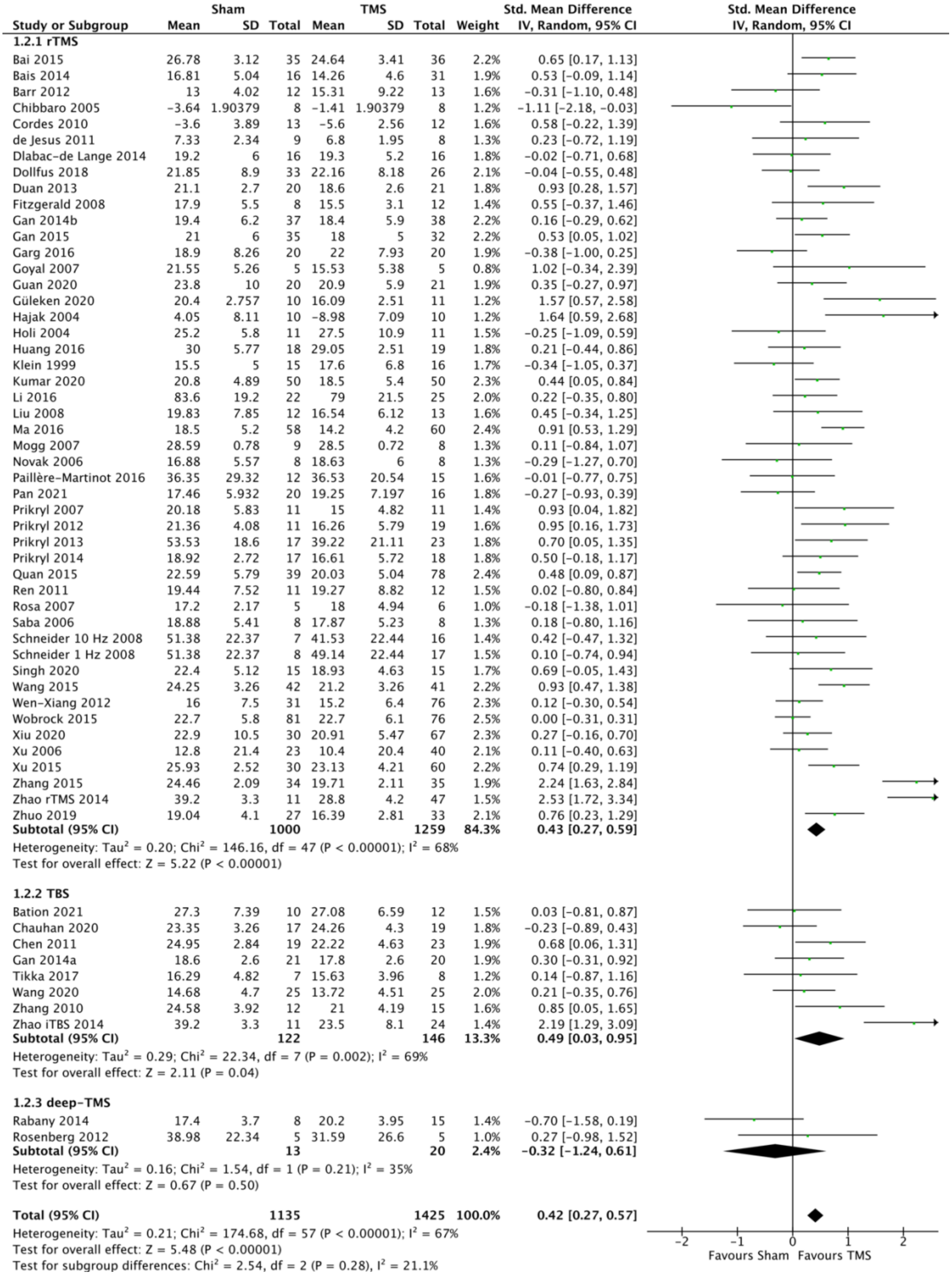

Zheng 2012 not included as the data were not reported separately for rTMS- and TBS-treated groups.

Supplementary Figure 3. Subgroup analysis stratified by treatment stimulation frequency (>1 Hz and 1 Hz). rTMS studies only.

	Sham			TMS				Std. Mean Difference
**Study or Subgroup**	**Mean**	**SD**	**Total**	**Mean**	**SD**	**Total**	**Weight**	**IV, Random, 95% CI**
**1.2.1 1 Hz**								
Bais 2014	16.81	5.04	16	14.26	4.6	31	2.3%	0.53 [−0.09, 1.14]
Chibbaro 2005	−3.64	1.90379	8	−1.41	1.90379	8	1.4%	−1.11 [−2.18, −0.03]
de Jesus 2011	7.33	2.34	9	6.8	1.95	8	1.6%	0.23 [−0.72, 1.19]
Klein 1999	15.5	5	15	17.6	6.8	16	2.0%	−0.34 [−1.05, 0.37]
Paillère-Martinot 2016	36.35	29.32	12	36.53	20.54	15	1.9%	−0.01 [−0.77, 0.75]
Rosa 2007	17.2	2.17	5	18	4.94	6	1.2%	−0.18 [−1.38, 1.01]
Saba 2006	18.88	5.41	8	17.87	5.23	8	1.5%	0.18 [−0.80, 1.16]
Schneider 1 Hz 2008	51.38	22.37	8	49.14	22.44	17	1.8%	0.10 [−0.74, 0.94]
Wen-Xiang 2012	16	7.5	31	15.2	6.4	76	2.7%	0.12 [−0.30, 0.54]
**Subtotal (95% CI)**			**112**			**185**	**16.4%**	**0.05 [−0.21, 0.30]**
Heterogeneity: $Tau^2 = 0.01$; $Chi^2 = 8.44$, df = 8 (P = 0.39); $I^2 = 5\%$								
Test for overall effect: Z = 0.35 (P = 0.72)								
**1.2.2 >1 Hz**								
Bai 2015	26.78	3.12	35	24.64	3.41	36	2.6%	0.65 [0.17, 1.13]
Barr 2012	13	4.02	12	15.31	9.22	13	1.9%	−0.31 [−1.10, 0.48]
Cordes 2010	−3.6	3.89	13	−5.6	2.56	12	1.8%	0.58 [−0.22, 1.39]
Dlabac-de Lange 2014	19.2	6	16	19.3	5.2	16	2.1%	−0.02 [−0.71, 0.68]
Dollfus 2018	21.85	8.9	33	22.16	8.18	26	2.5%	−0.04 [−0.55, 0.48]
Duan 2013	21.1	2.7	20	18.6	2.6	21	2.2%	0.93 [0.28, 1.57]
Fitzgerald 2008	17.9	5.5	8	15.5	3.1	12	1.6%	0.55 [−0.37, 1.46]
Gan 2014b	19.4	6.2	37	18.4	5.9	38	2.7%	0.16 [−0.29, 0.62]
Gan 2015	21	6	35	18	5	32	2.6%	0.53 [0.05, 1.02]
Garg 2016	18.9	8.26	20	22	7.93	20	2.2%	−0.38 [−1.00, 0.25]
Goyal 2007	21.55	5.26	5	15.53	5.38	5	1.0%	1.02 [−0.34, 2.39]
Guan 2020	23.8	10	20	20.9	5.9	21	2.3%	0.35 [−0.27, 0.97]
Güleken 2020	20.4	2.757	10	16.09	2.51	11	1.5%	1.57 [0.57, 2.58]
Hajak 2004	4.05	8.11	10	−8.98	7.09	10	1.4%	1.64 [0.59, 2.68]
Holi 2004	25.2	5.8	11	27.5	10.9	11	1.8%	−0.25 [−1.09, 0.59]
Huang 2016	30	5.77	18	29.05	2.51	19	2.2%	0.21 [−0.44, 0.86]
Kumar 2020	20.8	4.89	50	18.5	5.4	50	2.8%	0.44 [0.05, 0.84]
Li 2016	83.6	19.2	22	79	21.5	25	2.4%	0.22 [−0.35, 0.80]
Liu 2008	19.83	7.85	12	16.54	6.12	13	1.9%	0.45 [−0.34, 1.25]
Ma 2016	18.5	5.2	58	14.2	4.2	60	2.8%	0.91 [0.53, 1.29]
Mogg 2007	28.59	0.78	9	28.5	0.72	8	1.6%	0.11 [−0.84, 1.07]
Novak 2006	16.88	5.57	8	18.63	6	8	1.5%	−0.29 [−1.27, 0.70]
Pan 2021	17.46	5.932	20	19.25	7.197	16	2.2%	−0.27 [−0.93, 0.39]
Prikryl 2007	20.18	5.83	11	15	4.82	11	1.7%	0.93 [0.04, 1.82]
Prikryl 2012	21.36	4.08	11	16.26	5.79	19	1.9%	0.95 [0.16, 1.73]
Prikryl 2013	53.53	18.6	17	39.22	21.11	23	2.2%	0.70 [0.05, 1.35]
Prikryl 2014	18.92	2.72	17	16.61	5.72	18	2.1%	0.50 [−0.18, 1.17]
Quan 2015	22.59	5.79	39	20.03	5.04	78	2.8%	0.48 [0.09, 0.87]
Ren 2011	19.44	7.52	11	19.27	8.82	12	1.8%	0.02 [−0.80, 0.84]
Schneider 10 Hz 2008	51.38	22.37	7	41.53	22.44	16	1.7%	0.42 [−0.47, 1.32]
Singh 2020	22.4	5.12	15	18.93	4.63	15	2.0%	0.69 [−0.05, 1.43]
Wang 2015	24.25	3.26	42	21.2	3.26	41	2.7%	0.93 [0.47, 1.38]
Wobrock 2015	22.7	5.8	81	22.7	6.1	76	3.0%	0.00 [−0.31, 0.31]
Xiu 2020	22.9	10.5	30	20.91	5.47	67	2.7%	0.27 [−0.16, 0.70]
Xu 2015	25.93	2.52	30	23.13	4.21	60	2.7%	0.74 [0.29, 1.19]
Zhang 2015	24.46	2.09	34	19.71	2.11	35	2.3%	2.24 [1.63, 2.84]
Zhao rTMS 2014	39.2	3.3	11	28.8	4.2	47	1.8%	2.53 [1.72, 3.34]
Zheng 2012	22.8	5.1	17	23.02	5.14	56	2.4%	−0.04 [−0.59, 0.50]
Zhuo 2019	19.04	4.1	27	16.39	2.81	33	2.5%	0.76 [0.23, 1.29]
**Subtotal (95% CI)**			**882**			**1090**	**83.6%**	**0.51 [0.33, 0.69]**
Heterogeneity: $Tau^2 = 0.21$; $Chi^2 = 128.70$, df = 38 (P < 0.00001); $I^2 = 70\%$								
Test for overall effect: Z = 5.57 (P < 0.00001)								
**Total (95% CI)**			**994**			**1275**	**100.0%**	**0.43 [0.26, 0.59]**
Heterogeneity: $Tau^2 = 0.20$; $Chi^2 = 147.60$, df = 47 (P < 0.00001); $I^2 = 68\%$								
Test for overall effect: Z = 5.15 (P < 0.00001)								
Test for subgroup differences: $Chi^2 = 8.74$, df = 1 (P = 0.003), $I^2 = 88.6\%$								

Std. Mean Difference
IV, Random, 95% CI

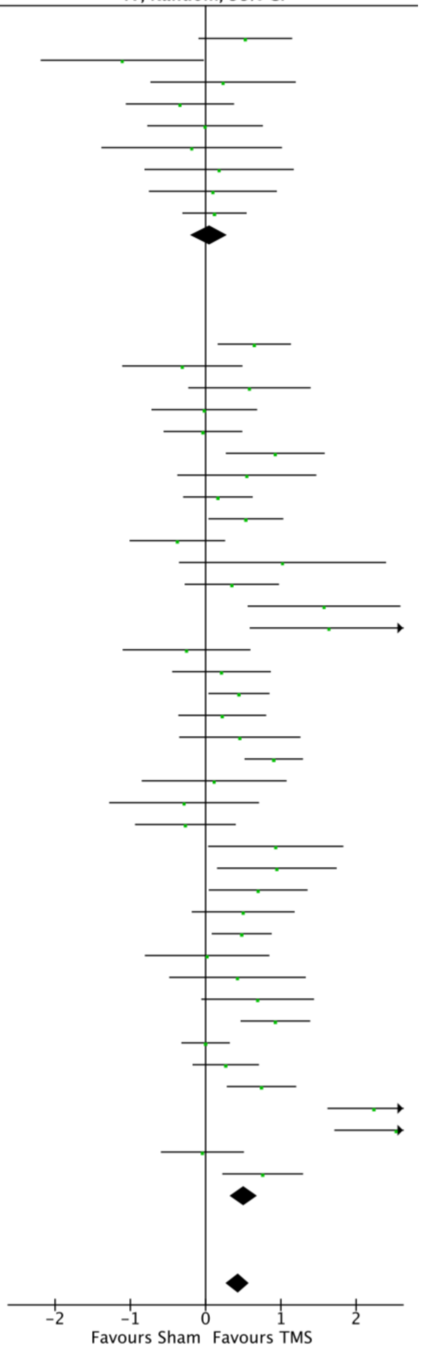

Supplementary Figure 4. Subgroup analysis stratified by treatment intensity (at or above 100% of motor threshold (MT) and under 100% of MT)

	Sham			TMS				Std. Mean Difference
**Study or Subgroup**	**Mean**	**SD**	**Total**	**Mean**	**SD**	**Total**	**Weight**	**IV, Random, 95% CI**
**1.2.1 < 100% MT**								
Bais 2014	16.81	5.04	16	14.26	4.6	31	2.0%	0.53 [−0.09, 1.14]
Barr 2012	13	4.02	12	15.31	9.22	13	1.5%	−0.31 [−1.10, 0.48]
Bation 2021	27.3	7.39	10	27.08	6.59	12	1.4%	0.03 [−0.81, 0.87]
Chauhan 2020	23.35	3.26	17	24.26	4.3	19	1.9%	−0.23 [−0.89, 0.43]
Chen 2011	24.95	2.84	19	22.22	4.63	23	2.0%	0.68 [0.06, 1.31]
Chibbaro 2005	−3.64	1.90379	8	−1.41	1.90379	8	1.0%	−1.11 [−2.18, −0.03]
de Jesus 2011	7.33	2.34	9	6.8	1.95	8	1.2%	0.23 [−0.72, 1.19]
Dlabac-de Lange 2014	19.2	6	16	19.3	5.2	16	1.8%	−0.02 [−0.71, 0.68]
Dollfus 2018	21.85	8.9	33	22.16	8.18	26	2.3%	−0.04 [−0.55, 0.48]
Güleken 2020	20.4	2.757	10	16.09	2.51	11	1.1%	1.57 [0.57, 2.58]
Ma 2016	18.5	5.2	58	14.2	4.2	60	2.8%	0.91 [0.53, 1.29]
Novak 2006	16.88	5.57	8	18.63	6	8	1.1%	−0.29 [−1.27, 0.70]
Quan 2015	22.59	5.79	39	20.03	5.04	78	2.8%	0.48 [0.09, 0.87]
Ren 2011	19.44	7.52	11	19.27	8.82	12	1.5%	0.02 [−0.80, 0.84]
Rosa 2007	17.2	2.17	5	18	4.94	6	0.9%	−0.18 [−1.38, 1.01]
Saba 2006	18.88	5.41	8	17.87	5.23	8	1.2%	0.18 [−0.80, 1.16]
Tikka 2017	16.29	4.82	7	15.63	3.96	8	1.1%	0.14 [−0.87, 1.16]
Wen-Xiang 2012	16	7.5	31	15.2	6.4	76	2.7%	0.12 [−0.30, 0.54]
Xu 2006	12.8	21.4	23	10.4	20.4	40	2.3%	0.11 [−0.40, 0.63]
Xu 2015	25.93	2.52	30	23.13	4.21	60	2.6%	0.74 [0.29, 1.19]
Zhang 2010	24.58	3.92	12	21	4.19	15	1.5%	0.85 [0.05, 1.65]
Zhao iTBS 2014	39.2	3.3	11	23.5	8.1	24	1.3%	2.19 [1.29, 3.09]
Zheng 2012	22.8	5.1	17	23.02	5.14	56	2.2%	−0.04 [−0.59, 0.50]
Zhuo 2019	19.04	4.1	27	16.39	2.81	33	2.3%	0.76 [0.23, 1.29]
**Subtotal (95% CI)**			**437**			**651**	**42.6%**	**0.33 [0.11, 0.55]**
Heterogeneity: $Tau^2 = 0.18$; $Chi^2 = 62.29$, df = 23 (P < 0.0001); $I^2 = 63\%$								
Test for overall effect: Z = 2.94 (P = 0.003)								
**1.2.2 = or > 100% MT**								
Bai 2015	26.78	3.12	35	24.64	3.41	36	2.5%	0.65 [0.17, 1.13]
Cordes 2010	−3.6	3.89	13	−5.6	2.56	12	1.5%	0.58 [−0.22, 1.39]
Duan 2013	21.1	2.7	20	18.6	2.6	21	1.9%	0.93 [0.28, 1.57]
Fitzgerald 2008	17.9	5.5	8	15.5	3.1	12	1.3%	0.55 [−0.37, 1.46]
Gan 2014a	18.6	2.6	21	17.8	2.6	20	2.0%	0.30 [−0.31, 0.92]
Gan 2014b	19.4	6.2	37	18.4	5.9	38	2.6%	0.16 [−0.29, 0.62]
Gan 2015	21	6	35	18	5	32	2.4%	0.53 [0.05, 1.02]
Garg 2016	18.9	8.26	20	22	7.93	20	2.0%	−0.38 [−1.00, 0.25]
Goyal 2007	21.55	5.26	5	15.53	5.38	5	0.7%	1.02 [−0.34, 2.39]
Guan 2020	23.8	10	20	20.9	5.9	21	2.0%	0.35 [−0.27, 0.97]
Hajak 2004	4.05	8.11	10	−8.98	7.09	10	1.1%	1.64 [0.59, 2.68]
Holi 2004	25.2	5.8	11	27.5	10.9	11	1.4%	−0.25 [−1.09, 0.59]
Huang 2016	30	5.77	18	29.05	2.51	19	1.9%	0.21 [−0.44, 0.86]
Klein 1999	15.5	5	15	17.6	6.8	16	1.7%	−0.34 [−1.05, 0.37]
Kumar 2020	20.8	4.89	50	18.5	5.4	50	2.8%	0.44 [0.05, 0.84]
Li 2016	83.6	19.2	22	79	21.5	25	2.1%	0.22 [−0.35, 0.80]
Liu 2008	19.83	7.85	12	16.54	6.12	13	1.5%	0.45 [−0.34, 1.25]
Mogg 2007	28.59	0.78	9	28.5	0.72	8	1.2%	0.11 [−0.84, 1.07]
Paillère-Martinot 2016	36.35	29.32	12	36.53	20.54	15	1.6%	−0.01 [−0.77, 0.75]
Pan 2021	17.46	5.932	20	19.25	7.197	16	1.9%	−0.27 [−0.93, 0.39]
Prikryl 2007	20.18	5.83	11	15	4.82	11	1.3%	0.93 [0.04, 1.82]
Prikryl 2012	21.36	4.08	11	16.26	5.79	19	1.5%	0.95 [0.16, 1.73]
Prikryl 2013	53.53	18.6	17	39.22	21.11	23	1.9%	0.70 [0.05, 1.35]
Prikryl 2014	18.92	2.72	17	16.61	5.72	18	1.8%	0.50 [−0.18, 1.17]
Rabany 2014	17.4	3.7	8	20.2	3.95	15	1.3%	−0.70 [−1.58, 0.19]
Rosenberg 2012	38.98	22.34	5	31.59	26.6	5	0.8%	0.27 [−0.98, 1.52]
Schneider 10 Hz 2008	51.38	22.37	7	41.53	22.44	16	1.3%	0.42 [−0.47, 1.32]
Schneider 1 Hz 2008	51.38	22.37	8	49.14	22.44	17	1.4%	0.10 [−0.74, 0.94]
Singh 2020	22.4	5.12	15	18.93	4.63	15	1.7%	0.69 [−0.05, 1.43]
Wang 2015	24.25	3.26	42	21.2	3.26	41	2.6%	0.93 [0.47, 1.38]
Wobrock 2015	22.7	5.8	81	22.7	6.1	76	3.1%	0.00 [−0.31, 0.31]
Xiu 2020	22.9	10.5	30	20.91	5.47	67	2.6%	0.27 [−0.16, 0.70]
**Subtotal (95% CI)**			**645**			**723**	**57.4%**	**0.35 [0.20, 0.50]**
Heterogeneity: $Tau^2 = 0.07$; $Chi^2 = 51.72$, df = 31 (P = 0.01); $I^2 = 40\%$								
Test for overall effect: Z = 4.61 (P < 0.00001)								
**Total (95% CI)**			**1082**			**1374**	**100.0%**	**0.35 [0.22, 0.47]**
Heterogeneity: $Tau^2 = 0.11$; $Chi^2 = 114.26$, df = 55 (P < 0.00001); $I^2 = 52\%$								
Test for overall effect: Z = 5.39 (P < 0.00001)								
Test for subgroup differences: $Chi^2 = 0.02$, df = 1 (P = 0.89), $I^2 = 0\%$								

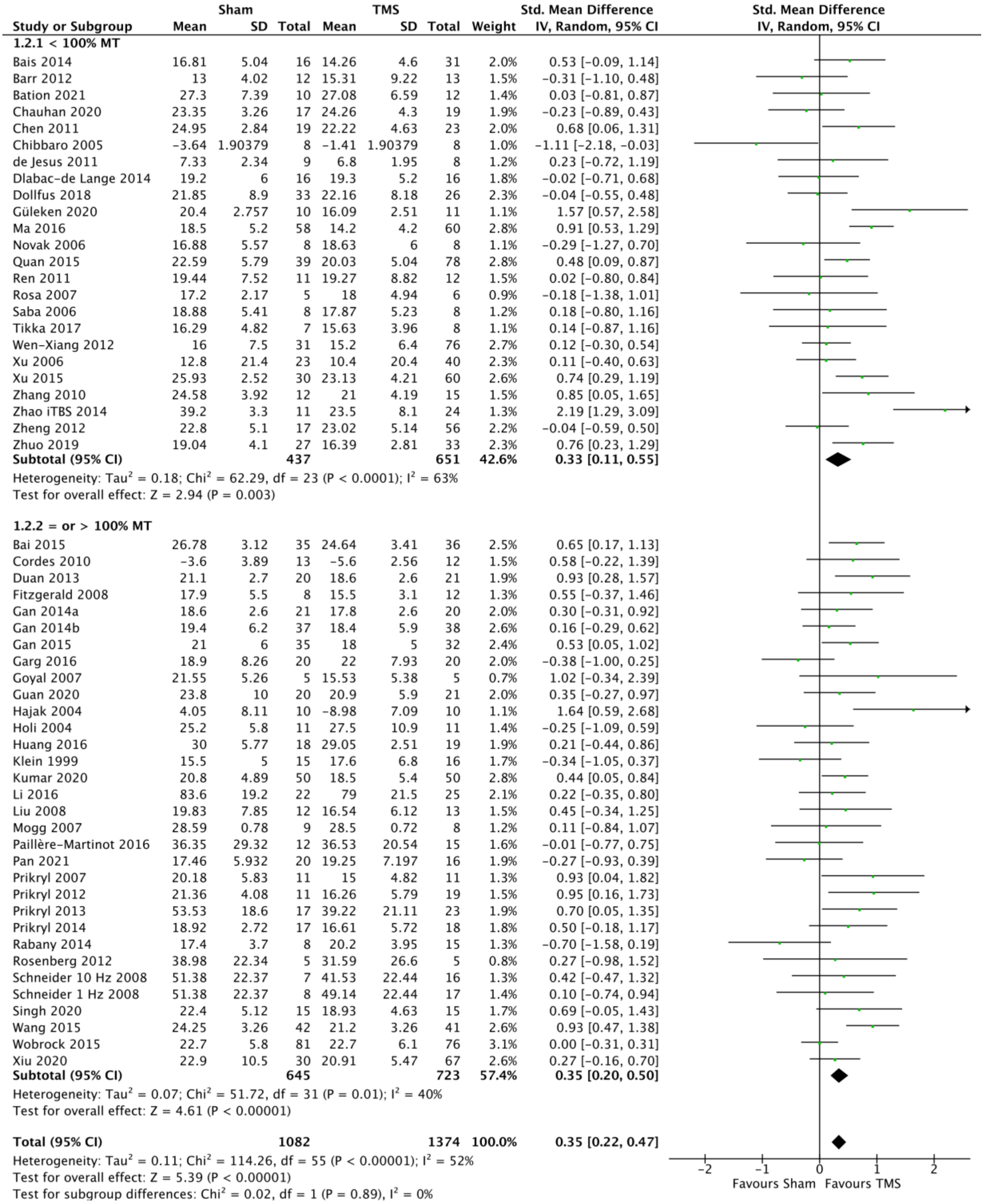

Data not available for Wang 2015 and Zhang 2015. Zhao rTMS 2014 used a variable intensity (80-110%) and was not included.

Supplementary Figure 5. Subgroup analysis stratified by age (at or below median age (35.5 years) and above median age)

Study or Subgroup	Sham Mean	Sham SD	Sham Total	TMS Mean	TMS SD	TMS Total	Weight	Std. Mean Difference IV, Random, 95% CI
**1.2.1 = or < median age (35.5 years)**								
Bai 2015	26.78	3.12	35	24.64	3.41	36	2.2%	0.65 [0.17, 1.13]
Bation 2021	27.3	7.39	10	27.08	6.59	12	1.5%	0.03 [−0.81, 0.87]
Cordes 2010	−3.6	3.89	13	−5.6	2.56	12	1.6%	0.58 [−0.22, 1.39]
Dlabac-de Lange 2014	19.2	6	16	19.3	5.2	16	1.8%	−0.02 [−0.71, 0.68]
Duan 2013	21.1	2.7	20	18.6	2.6	21	1.9%	0.93 [0.28, 1.57]
Fitzgerald 2008	17.9	5.5	8	15.5	3.1	12	1.4%	0.55 [−0.37, 1.46]
Gan 2014a	18.6	2.6	21	17.8	2.6	20	2.0%	0.30 [−0.31, 0.92]
Gan 2014b	19.4	6.2	37	18.4	5.9	38	2.3%	0.16 [−0.29, 0.62]
Gan 2015	21	6	35	18	5	32	2.2%	0.53 [0.05, 1.02]
Garg 2016	18.9	8.26	20	22	7.93	20	1.9%	−0.38 [−1.00, 0.25]
Goyal 2007	21.55	5.26	5	15.53	5.38	5	0.9%	1.02 [−0.34, 2.39]
Güleken 2020	20.4	2.757	10	16.09	2.51	11	1.3%	1.57 [0.57, 2.58]
Klein 1999	15.5	5	15	17.6	6.8	16	1.8%	−0.34 [−1.05, 0.37]
Liu 2008	19.83	7.85	12	16.54	6.12	13	1.6%	0.45 [−0.34, 1.25]
Novak 2006	16.88	5.57	8	18.63	6	8	1.3%	−0.29 [−1.27, 0.70]
Paillère-Martinot 2016	36.35	29.32	12	36.53	20.54	15	1.7%	−0.01 [−0.77, 0.75]
Prikryl 2007	20.18	5.83	11	15	4.82	11	1.5%	0.93 [0.04, 1.82]
Prikryl 2012	21.36	4.08	11	16.26	5.79	19	1.6%	0.95 [0.16, 1.73]
Prikryl 2013	53.53	18.6	17	39.22	21.11	23	1.9%	0.70 [0.05, 1.35]
Prikryl 2014	18.92	2.72	17	16.61	5.72	18	1.8%	0.50 [−0.18, 1.17]
Rabany 2014	17.4	3.7	8	20.2	3.95	15	1.5%	−0.70 [−1.58, 0.19]
Ren 2011	19.44	7.52	11	19.27	8.82	12	1.6%	0.02 [−0.80, 0.84]
Rosa 2007	17.2	2.17	5	18	4.94	6	1.0%	−0.18 [−1.38, 1.01]
Saba 2006	18.88	5.41	8	17.87	5.23	8	1.3%	0.18 [−0.80, 1.16]
Singh 2020	22.4	5.12	15	18.93	4.63	15	1.7%	0.69 [−0.05, 1.43]
Tikka 2017	16.29	4.82	7	15.63	3.96	8	1.3%	0.14 [−0.87, 1.16]
Wobrock 2015	22.7	5.8	81	22.7	6.1	76	2.6%	0.00 [−0.31, 0.31]
Zhuo 2019	19.04	4.1	27	16.39	2.81	33	2.1%	0.76 [0.23, 1.29]
**Subtotal (95% CI)**			**495**			**531**	**47.2%**	**0.34 [0.17, 0.51]**
Heterogeneity: $Tau^2 = 0.08$; $Chi^2 = 45.63$, df = 27 (P = 0.01); $I^2 = 41\%$								
Test for overall effect: Z = 3.84 (P = 0.0001)								
**1.2.2 >median age (35.5 years)**								
Bais 2014	16.81	5.04	16	14.26	4.6	31	2.0%	0.53 [−0.09, 1.14]
Barr 2012	13	4.02	12	15.31	9.22	13	1.6%	−0.31 [−1.10, 0.48]
Chauhan 2020	23.35	3.26	17	24.26	4.3	19	1.9%	−0.23 [−0.89, 0.43]
Chen 2011	24.95	2.84	19	22.22	4.63	23	1.9%	0.68 [0.06, 1.31]
Chibbaro 2005	−3.64	1.90379	8	−1.41	1.90379	8	1.2%	−1.11 [−2.18, −0.03]
de Jesus 2011	7.33	2.34	9	6.8	1.95	8	1.3%	0.23 [−0.72, 1.19]
Dollfus 2018	21.85	8.9	33	22.16	8.18	26	2.2%	−0.04 [−0.55, 0.48]
Guan 2020	23.8	10	20	20.9	5.9	21	1.9%	0.35 [−0.27, 0.97]
Hajak 2004	4.05	8.11	10	−8.98	7.09	10	1.2%	1.64 [0.59, 2.68]
Holi 2004	25.2	5.8	11	27.5	10.9	11	1.5%	−0.25 [−1.09, 0.59]
Huang 2016	30	5.77	18	29.05	2.51	19	1.9%	0.21 [−0.44, 0.86]
Kumar 2020	20.8	4.89	50	18.5	5.4	50	2.4%	0.44 [0.05, 0.84]
Li 2016	83.6	19.2	22	79	21.5	25	2.0%	0.22 [−0.35, 0.80]
Mogg 2007	28.59	0.78	9	28.5	0.72	8	1.4%	0.11 [−0.84, 1.07]
Pan 2021	17.46	5.932	20	19.25	7.197	16	1.9%	−0.27 [−0.93, 0.39]
Quan 2015	22.59	5.79	39	20.03	5.04	78	2.4%	0.48 [0.09, 0.87]
Rosenberg 2012	38.98	22.34	5	31.59	26.6	5	1.0%	0.27 [−0.98, 1.52]
Schneider 10 Hz 2008	51.38	22.37	7	41.53	22.44	16	1.4%	0.42 [−0.47, 1.32]
Schneider 1 Hz 2008	51.38	22.37	8	49.14	22.44	17	1.5%	0.10 [−0.74, 0.94]
Wang 2015	24.25	3.26	42	21.2	3.26	41	2.3%	0.93 [0.47, 1.38]
Wen-Xiang 2012	16	7.5	31	15.2	6.4	76	2.4%	0.12 [−0.30, 0.54]
Xiu 2020	22.9	10.5	30	20.91	5.47	67	2.3%	0.27 [−0.16, 0.70]
Xu 2006	12.8	21.4	23	10.4	20.4	40	2.2%	0.11 [−0.40, 0.63]
Xu 2015	25.93	2.52	30	23.13	4.21	60	2.3%	0.74 [0.29, 1.19]
Zhang 2010	24.58	3.92	12	21	4.19	15	1.6%	0.85 [0.05, 1.65]
Zhang 2015	24.46	2.09	34	19.71	2.11	35	2.0%	2.24 [1.63, 2.84]
Zhao iTBS 2014	39.2	3.3	11	23.5	8.1	24	1.4%	2.19 [1.29, 3.09]
Zhao rTMS 2014	39.2	3.3	11	28.8	4.2	47	1.6%	2.53 [1.72, 3.34]
Zheng 2012	22.8	5.1	17	23.02	5.14	56	2.1%	−0.04 [−0.59, 0.50]
**Subtotal (95% CI)**			**574**			**865**	**52.8%**	**0.46 [0.22, 0.70]**
Heterogeneity: $Tau^2 = 0.32$; $Chi^2 = 122.19$, df = 28 (P < 0.00001); $I^2 = 77\%$								
Test for overall effect: Z = 3.70 (P = 0.0002)								
**Total (95% CI)**			**1069**			**1396**	**100.0%**	**0.40 [0.25, 0.56]**
Heterogeneity: $Tau^2 = 0.21$; $Chi^2 = 169.98$, df = 56 (P < 0.00001); $I^2 = 67\%$								
Test for overall effect: Z = 5.17 (P < 0.00001)								
Test for subgroup differences: $Chi^2 = 0.63$, df = 1 (P = 0.43), $I^2 = 0\%$								

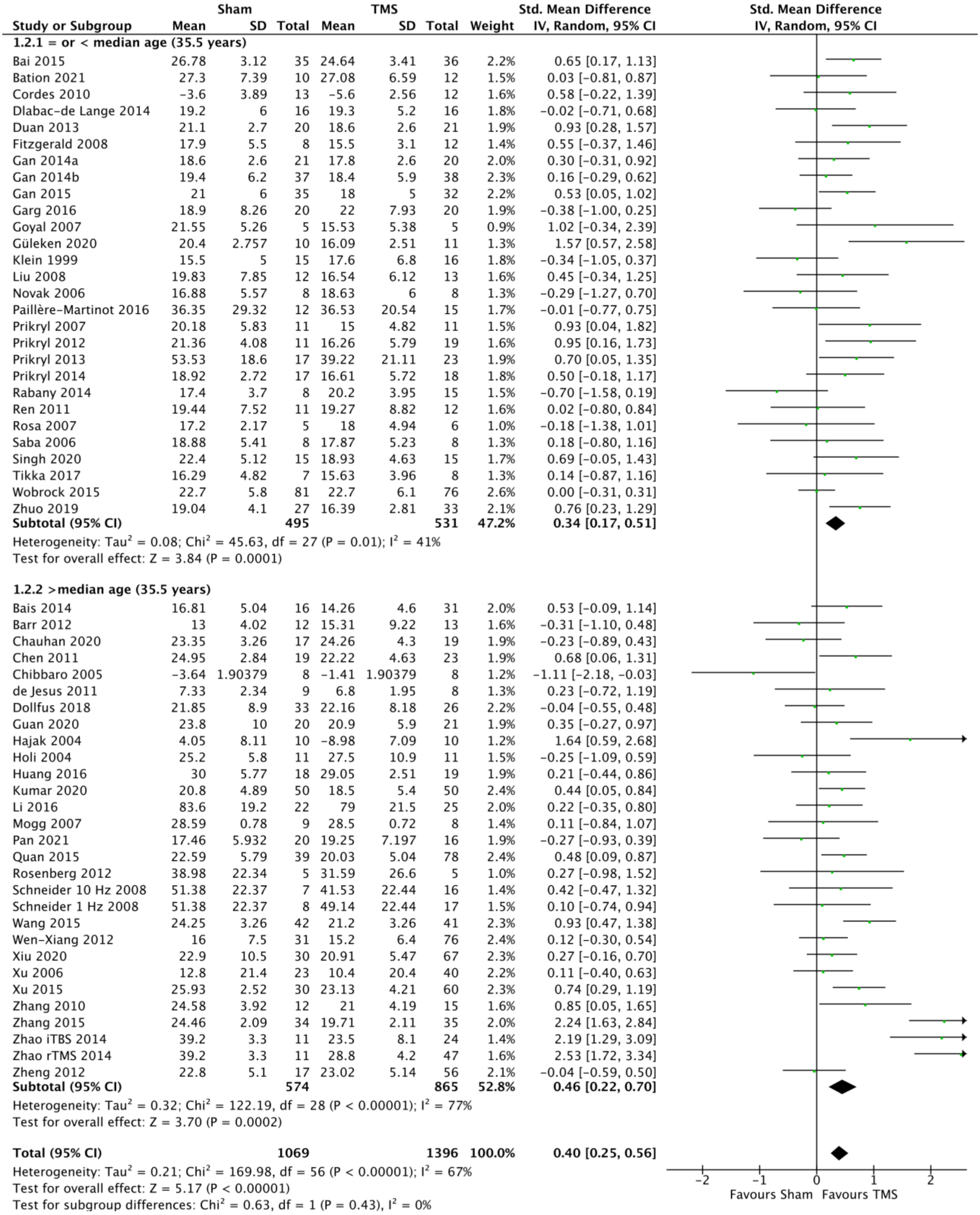

Data not available for Ma 2016 and Wang 2020.
